# Interhemispheric integration in the neural face perception network: Does stimulus location matter?

**DOI:** 10.1162/IMAG.a.17

**Published:** 2025-05-29

**Authors:** Julia Elina Stocker, Antonia Schulz, Ina Thome, Jens Sommer, Jonas Rabeneck, Kristin Marie Rusch, Olaf Steinsträter, Andreas Jansen

**Affiliations:** Department of Psychiatry and Psychotherapy, University of Marburg, Marburg, Germany; Core-Facility Brainimaging, Faculty of Medicine, University of Marburg, Marburg, Germany; Department of Neurology, University of Freiburg, Freiburg, Germany; Center for Mind, Brain and Behavior (CMBB), Universities of Marburg, Gießen and Darmstadt, Germany

**Keywords:** face perception, lateralization, network, OFA, FFA, interhemispheric transfer, DCM, fMRI, peripheral stimulation

## Abstract

The neural mechanisms underlying hemispheric lateralization can be investigated using neuroimaging methods and modelling techniques. In some experiments, sensory information is initially presented exclusively to one hemisphere, for example, by displaying a visual stimulus in the periphery of the contralateral hemifield. This experimental design enables, among other things, a comparison of competing theories of interhemispheric integration (e.g., interhemispheric inhibition vs. interhemispheric recruitment). However, the underlying neural models for peripheral stimulation may differ from those for central stimulation and, therefore, may not adequately describe the mechanisms associated with typical, foveal stimulus processing. To address this question, the present functional magnetic resonance imaging (fMRI) study analysed the influence of stimulus location (peripheral vs. central) on neural network connectivity, particularly interhemispheric transfer, as determined by dynamic causal modelling (DCM), for a face perception task. Face and object images were presented either peripherally or centrally to a group of healthy volunteers (*N*= 17). By contrasting brain activations for faces against objects, we identified bilateral face-sensitive regions, such as the left and right fusiform face area (FFA) and the occipital face area (OFA). Additionally, we extracted the bilateral primary visual cortex (V1) as the input region for our neural models. We constructed five increasingly complex models that differed only in their modulatory connectivity. Bayesian model averaging (BMA) was employed to average the parameters across all models, enabling the calculation of interhemispheric transfer difference (i.e., left-to-right minus right-to-left modulatory connectivity parameter) and the strength of interhemispheric transfer between bilateral OFA and FFA regions. Our findings demonstrate that interhemispheric integration depends on stimulus location. Peripheral presentations of faces induce different connectivity patterns compared with centrally depicted faces. Specifically, we observed larger interhemispheric transfer differences for peripheral face stimuli compared with central stimuli. In conclusion, peripheral and central presentations of faces modulate the face processing network differently, with left and right visual field presentations yielding asymmetrical connectivity patterns. Since faces are preferentially processed via the fovea, the typical face processing network likely aligns more closely with activation patterns elicited by central stimuli. In contrast, connectivity patterns triggered by peripheral stimulation may represent an atypical processing style and cannot be directly compared with those activated by central stimuli.

## 1 Introduction

The neural network for face processing is often divided into a “core system” and an “extended system” (Haxby et al., 2000). According toHaxby et al. (2000), the core system consists of three bilateral brain regions in the occipito-temporal cortex. These regions are the occipital face area (OFA) in the inferior occipital gyrus, the fusiform face area (FFA) in the lateral fusiform gyrus, and a face-sensitive region in the posterior superior temporal sulcus (pSTS). The extended system consists of functionally less face-specific brain areas in limbic, parietal, and prefrontal regions. Depending on the specific task requirements, these regions are recruited to extract further information from faces, for instance, the emotional state or the trustworthiness of a person (Haxby et al., 2000;Duchaine & Yovel 2015).

Although the face processing network is, at least on the population level, right-dominant, the brain regions are at first glance distributed mirror-symmetrically across both hemispheres (Thome et al., 2022). OFA, FFA, and pSTS, therefore, exist in the left and right hemisphere. While there are now hundreds of studies that investigated functional differences between face processing areas in the posterior-anterior direction within one hemisphere (e.g., right OFA vs. right FFA), there are much fewer studies that explicitly assessed functional differences between left and right hemispheric homologues (e.g., left FFA vs. right FFA). One possible reason is that these differences are smaller than between regions within a hemisphere.Davies-Thompson and Andrews (2012)showed, for example, that the functional connectivity (operationalized by the residual time courses after stimulus-driven activity was removed) between homologous face regions in different hemispheres is higher than between different face regions in the same hemisphere. Studies that explicitly investigated differences between homologous face regions most often focused on the FFA.Verosky and Turk-Browne (2012)investigated the hypothesis that the left hemisphere can recognize faces only with support from the right hemisphere. They presented faces in the left or right visual field and showed that face identity adaptation in the left FFA occurred only when a face had previously been processed by the right hemisphere, but not when it had only been processed by the left hemisphere.Ma and Han (2012)investigated the functional role of the FFA in high-level social cognition using morphs between self-face and a gender-matched friend’s face. They showed that left FFA activity was associated with self-face physical properties, while right FFA activity was associated with self-face identity.Rossion et al. (2000)investigated FFA activity for processing faces as a whole and for processing faces based on face features. They found that the right FFA was more activated when matching whole faces than face parts, whereas this activity pattern was reversed in the left homologous region. Meng et al. investigated the effect of face semblance (Meng et al., 2012). They reported that activity in the left FFA correlated with physical image-level semblance, while right FFA activity was associated with categorical face/no-face decisions. Taken together, these studies might indicate that the left FFA is more strongly associated with the processing of basic physical stimulus properties, while the right FFA is related to higher level processes. However, there is no overall neuroanatomical model of face processing that assigns specific cognitive functions to one or the other hemisphere, in particular for other regions than the FFA.

There is also a lack of detailed network models that describe how the two hemispheres interact with each other. Since both hemispheres are typically implicated in face processing, an exchange of information between the two hemispheres is necessary. This exchange most likely takes place primarily via homotopic connections (e.g., the left OFA—right OFA connection), not via the structurally weaker heterotopic connections (e.g., the left OFA—right FFA connection) (Catani & Thiebaut de Schotten, 2008;Hofer & Frahm, 2006). So far, it is not known how the hemispheres interact and in particular at what level of abstraction information is exchanged. The interaction between the hemispheres during face processing can be described using imaging methods and suitable modelling techniques. A particularly suitable modelling technique is dynamic causal modelling (DCM) (Friston et al., 2003). DCM describes how experimental manipulations (e.g., task demands) perturb the neural state of brain regions and the directed interactions among those regions. In its original implementation, DCM rests on a bilinear differential equation (i.e., low-order Taylor series approximation) to model the neural dynamics of the brain regions under consideration. For functional magnetic resonance imaging (fMRI) data, a haemodynamic model translates the predicted neural dynamics into a blood oxygenation level dependent (BOLD) signal time series, which can be compared with the acquired fMRI data. Hence, the bilinear neural state equation and the haemodynamic model jointly form a multiple-input multiple-output (MIMO) system in which the experimental manipulations (input) are deterministically linked to the measured BOLD signal in the brain regions (output). This MIMO system can be inverted (e.g., by using a Variational Bayes (VB) optimization scheme with Gaussian assumptions on the prior and posterior distributions;Zeidman et al., 2023) to compare different neural models and to calculate the posterior densities of the model parameters (i.e., conditional mean and variance).

In recent years, DCM has been used in various studies to investigate interhemispheric interactions (Chen et al., 2009;Frässle et al., 2016a,2016b,2016c;Grefkes et al., 2008;Lee et al., 2022;Seghier et al., 2011;Stephan et al., 2005;Stephan, Marshall, et al., 2007). In some studies, the visual stimuli were not presented centrally, but in the right or left hemifield (Frässle et al., 2016a,2016b,2016c;Stephan et al., 2005;Stephan, Marshall, et al., 2007, for an overview seeStephan, Fink, et al., 2007). Peripheral stimulus presentation does not activate the primary visual cortex in both hemispheres of the brain, as is the case with foveal presentation. It only activates the contralateral hemisphere due to the distinct topography of the neuronal fibres from the retina to the visual cortex. The stimulus information subsequently must be transmitted through the corpus callosum (or some alternative connection, e.g., subcortical pathways) before it can be processed by the hemisphere ipsilateral to stimulus presentation. Peripheral stimulus presentations can in particular be used to compare different interhemispheric processing mechanisms (e.g., interhemispheric inhibition vs. interhemispheric recruitment,Stephan, Marshall, et al., 2007), whereby it might possibly provide new insights into the lateralization of brain functions. In previous studies (Frässle et al., 2016a,2016b,2016c), our research group applied such an experimental design to analyse the connectivity between core regions of the face perception network (i.e., left and right OFA, left and right FFA) to unveil the mechanisms underlying both intra- and interhemispheric integration. Our results suggested that the right-hemispheric lateralization of the face network was associated with an asymmetric face-specific interhemispheric connectivity difference between the left and right OFA. At the structural level, this connectivity difference was correlated with grey matter volume in the OFA. At the physiological level, the connectivity difference was correlated with the strength of pupil constriction in response to faces, a measure with potential sensitivity to holistic (as opposed to feature-based) processing of faces (Frässle et al., 2016a).

When interpreting the DCM results, however, it must be considered that the interhemispheric coupling can be altered by peripheral presentation of information because sensory visual information enters just one hemisphere, not both hemispheres simultaneously. The neural model which is derived from a DCM analysis for an experimental design may, therefore, be different for a peripheral compared with a foveal stimulus presentation. While DCM analyses of data acquired using an experimental design with peripheral stimulus presentations are, therefore, well suited to assess different models of interhemispheric integration, they do not necessarily describe the neural mechanisms associated with typical, foveal stimulus presentation and, therefore, only allow limited conclusions about how the brain typically processes foveal information. To investigate this question, in the present fMRI study, we analyzed the influence of stimulus location (peripheral vs. central) on neural network connectivity as determined by DCM. We used a face-processing task in which stimuli were presented either at the center or periphery (i.e., left or right) of the visual field. In particular, we assessed whether the peripheral presentation of a stimulus leads to an increased interhemispheric transfer between the OFAs and the FFAs compared with a central presentation (as described by the face-specific modulatory connectivity). The changes in interhemispheric transfer were operationalized in two ways. First, we calculated the change in the interhemispheric transfer difference by the difference of the left-to-right and the right-to-left modulatory connectivity parameters. This variable describes whether the relative transfer across the hemispheres changes, for example, by increasing or decreasing the overall transfer from one hemisphere to the other or by reversing the direction. Second, we calculated the changes in the overall interhemispheric transfer strength by the average of the absolute value of the left-to-right modulatory connectivity parameter and the absolute value of the right-to-left modulatory connectivity parameter. This variable describes whether the absolute transfer across the hemispheres increases or decreases.

## 2 Methods

### 2.1 Participants

Twenty participants (10 female, 9 male, 1 non-binary; age range 18−40 years, mean age 26 years) were included in the study. Exclusion criteria were a history of drug and alcohol abuse, non-right-handedness (as assessed by the Edinburgh Handedness Inventory, (Oldfield, 1971), cut-off +/-30), medical contraindications against an MRI examination and pregnancy. The mean handedness quotient was 85.85 (std = 20.20). All participants gave their informed consent prior to the study. As compensation for their time, they received 20 € for their participation. The study procedure conformed to the Declaration of Helsinki and was approved by the local ethics committee of the Medical Faculty of the University of Marburg (file ref. 23–75 BO). The data of one participant (sub-02) had to be excluded from the analysis due to data quality problems. More specifically, the registration between the anatomical and functional images was of low quality, as a standardized visual inspection revealed.

The required sample size was calculated by a power analysis using G*Power (version 3.1,Faul et al., 2009). The expected effect size was calculated by the average outcome from previous study results (Frässle et al., 2016a;Maleki, 2021). As main analysis, we investigated the influences of central and peripheral stimulus presentation (paired t-test) on the directed interhemispheric transfer between the right and left OFA and the right and left FFA. The statistical power was set to 0.95, the alpha error estimate to 0.0125 (nominal*p*= 0.05), 4 tests for transfer between OFA and FFA. With the calculated Cohens’d of 1.17, we yielded a sample size of 16 participants. As we expected to exclude up to 30% of the participants due to poor data quality, we decided to measure 20 participants.

### 2.2 Experimental procedure

Before starting data acquisition, we preregistered our study on the online platform Open Science Framework (OSF) under the following URL:https://doi.org/10.17605/OSF.IO/T9YM6.

**Fig. 1.**
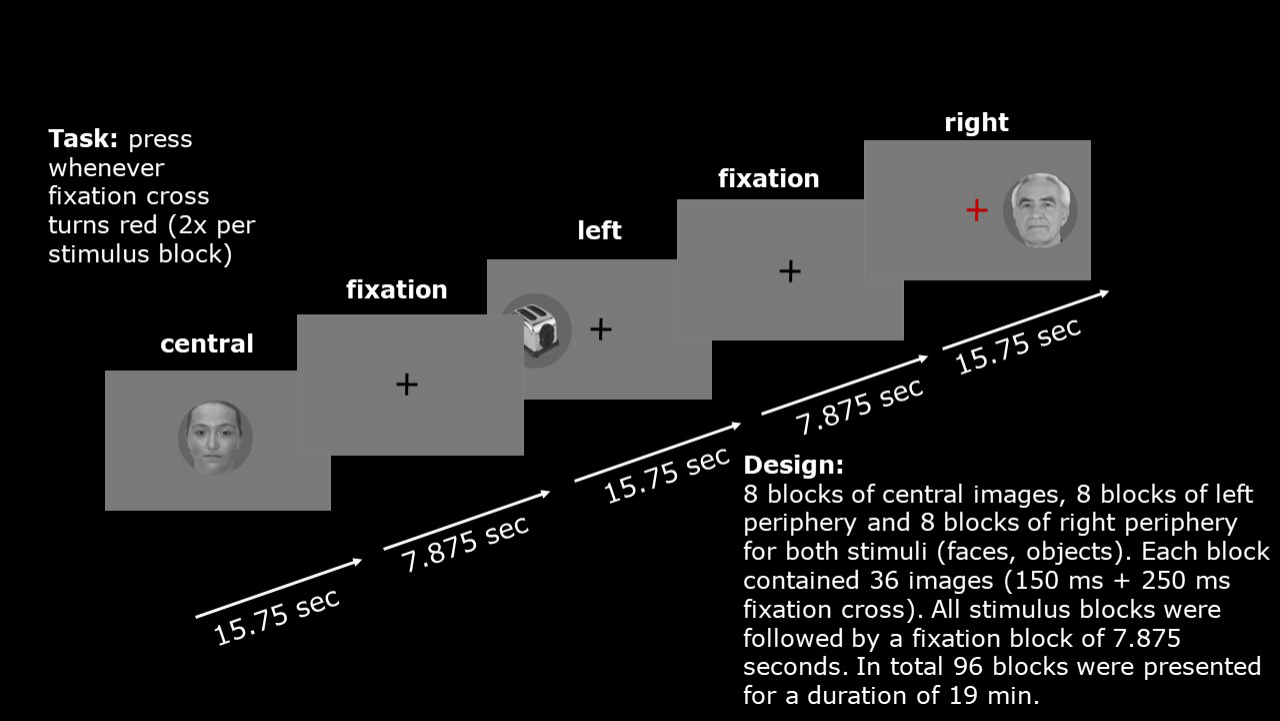
Graphical depiction of the experimental task. Subjects were presented with blocks of faces or non-face objects. The stimuli were presented either at the center or periphery (left or right) of the visual field.

We collected fMRI data during a face perception task in a 2 x 3 factorial, pseudo-randomized block design (Fig. 1). Participants viewed grey-scaled photographs of either neutral, white faces (F), or objects (O) (factor 1: stimulus class). The stimuli were presented either at the center (C) or periphery (L/R) of the visual field (factor 2: visual field). All pictures were presented in circular patches with 4.34° diameter on an MRI-compatible LCD screen (60 Hz, 4:3, 1024 x 786 pix). Importantly, the peripheral stimuli were centered at a visual angle of 4.02° to the left (L) or right (R) (Frässle et al., 2016a). The face stimuli were retrieved from the Center for Vital Longevity face Database (Ebner, 2008) with an equal female-to-male and young-to-old ratio. In total, we used 40 different face and object stimuli, respectively.

The face perception task was programmed using the Presentation® software (*Presentation*, 2023). Hereby, all six conditions (FL, FR, FC, OL OR, OC) for both stimulus types were presented in the eight blocks (i.e., 48 blocks in total). Each block contained 36 images. Each image was presented for 150 ms, followed by an ISI (inter-stimulus interval) of 250 ms, where only a fixation cross was visible before the next image appeared. One block lasted for 15.75 seconds. All blocks were separated by a low-level baseline period where only the fixation cross was shown. This baseline period lasted half as long as the face or object block. In total, participants were presented with 96 blocks (24 face blocks, 24 object blocks, 48 baseline blocks). The whole experiment lasted about 23 minutes. Before the start of the experiment, participants were instructed to always look at the fixation cross and to press a button whenever the fixation cross turned red. This happened twice in each face and object block at random time points, but not in the low-level baseline blocks. The fixation was controlled via online eye tracking at a rate of 500 Hz using an MRI-compatible infrared-sensitive camera (EyeLink 1000, SR Research, Osgoode, ON, Canada).

### 2.3 Image acquisition

Participants were scanned on a 3-Tesla MR scanner (Siemens TIM Trio, Erlangen Germany) with a 12-channel head matrix receive coil at the Department of Psychiatry of the University of Marburg. FMRI data were collected using a T2*-weighted single-shot gradient-echo echo-planar imaging sequence (EPI) (30 slices, 4 mm slice thickness, repetition time (TR) = 1450 ms, echo time (TE) = 25 ms, matrix size = 64 × 64 voxels, voxel size = 3 × 3 × 4 mm^2^, field of view (FoV) = 192 × 192 mm^2^, flip angle (FA) = 90°). Slices were measured in descending order parallel to the inter-commissural (AC-PC) plane. Images were acquired continuously, not interleaved, across the brain. The continuous acquisition reduces time residuals, which is an important aspect of a DCM analysis (Stephan et al., 2010). The measurement volume covered the whole brain. In total, 800 functional images were collected. Before the analysis, the first five images were discarded. Additionally, a high-resolution anatomical image was acquired using a T1-weighted rapid gradient-echo sequence in the sagittal plane (176 slices, 1 mm thickness, TR = 1900 ms, TE = 2.26 ms, matrix size = 256 × 256 voxels, voxel size = 1 × 1 × 1 mm^3^, FoV = 256 × 256 mm^2^, FA = 9°). The acquisition time of the anatomical scan was 4 minutes.

### 2.4 Data processing

MRI analysis was performed with the SPM12 (v7771) software package (Friston et al., 2014) in Matlab2021 (The MathWorks, Inc., 2022), using standard routines and default values as suggested by Canlab (Cognitive and Affective Neuroscience Laboratory, 2023). Before preprocessing, functional images were transformed into the Brain Imaging Data Structure (BIDS) and image quality was checked using the MRIQC pipeline (Esteban et al., 2017), see the Supplementary Section,Table S2for more details.

#### 2.4.1 Preprocessing

Preprocessing was performed in the following order: motion correction, slice time correction, coregistration with the anatomical image, normalization to MNI152 coordinate space, using the unified segmentation-normalization approach (Ashburner & Friston, 2005), and smoothing. Motion estimation was performed using a six-parameter-based, affine rigid-body transformation, adjusting the images to their mean via SPM’s 4*^th^*degree B-spline interpolation without any additional wrapping for all images and the mean. We corrected for descending slice time acquisition of the single volumes with SPM’s Fourier phase shift algorithm, using the middle slice (*n*= 15) as a reference. Further, we estimated the coregistration of the anatomical scan to our motion-corrected mean functional image. Thereby, we again choose SPM’s standardized interpolation method, optimized by the normalized mutual information criterion. Afterwards, we normalized all corrected functional images to SPM’s MNI 2 x 2 x 2 mm^3^template. SPM uses a mutual information, 12-parametric, affine regularization for estimation and transforms the images via a 4*^th^*degree B-Spline interpolation, whereby we selected voxel sizes of [2 2 2]. In the last step, we smoothed the images with an isotropic kernel of 6 mm to reduce noise in the individual samples (Friston et al., 2007).

#### 2.4.2 Statistical analysis

Statistical analysis of the preprocessed data was conducted using a General Linear Model (GLM). At the subject level, we included the following conditions as regressors: face central (FC), face left (FL), face right (FR), object central (OC), object left (OL), and object right (OR). The model further included six realignment parameters to account for linear movement artefacts, as well as the default high-pass filter that removes signals with periods longer than 128 seconds. We created*t*-statistic maps of the “faces > objects” (F > O) contrast to show brain activation in face-related regions (in particular the FFA and OFA). We used the “faces and objects > baseline” (F+ O > B) contrast to show activation in the primary visual cortex (V1). At the group level, we applied a one-sample*t*-test to calculate brain activation across participants. Here, we used the same contrasts for the second-level analysis.

#### 2.4.3 Determination of ROIs

To investigate the effective connectivity within the core face perception network, we defined six regions of interest (ROIs): the left and right V1, the left and right OFA, and the left and right FFA (see below). Since the exact localization of these regions varies between subjects, we determined the ROIs individually for each subject. First, we determined the group activation maxima for each ROI at a threshold of*p*= 0.001 (uncorrected for multiple comparisons). For the FFAs and OFAs, we used the “F > O” contrast. We determined the group activation maxima as the coordinates with the highest t-values within spherical masks (6 mm radius, dilation of one) centered at coordinates from the Thome et al. study (Thome et al., 2022). For the V1s, we used the “F + O > B” contrast. Here, we determined the maxima within anatomical masks encompassing left and right Brodmann area 17, respectively (WFU-Pickatlas, 2D dilation: 1 voxel in each direction,Maldjian et al., 2003,^2004^). Second, we retrieved individual coordinates for each ROI. For that, we determined the global activation maximum in each subject-specific activity map thresholded at*p*= 0.001 (uncorrected) and a cluster extent threshold of zero within a sphere with 15 mm radius centred at the group-level maximum for the specific ROI. If it was not possible to find activation at this threshold, we adjusted the activity map threshold to*p*= 0.01 or*p*= 0.1 (uncorrected), as previously suggested (e.g.,Zeidman et al., 2019;Rossion et al., 2012). If it was not possible, even at these liberal thresholds, to determine individual activation maxima for all six ROIs, the participant’s data were excluded from the DCM analysis. Afterwards, we inspected visually whether the obtained individual maximum was associated correctly with the region’s presumed location. If it became obvious that another local maximum than the automatically retrieved better fits a specific ROI, we manually adjusted these coordinates (seeSupplementary Table S4).

#### 2.4.4 Extraction of time series

For each ROI, we extracted the BOLD signal time series as the first eigenvariate of the voxels within a 6 mm radius around subject-specific ROI center coordinates (*p*< 0.001 uncorr.). The time series were mean centred and movement-related variance was removed (by adjustment concerning an effects-of-interest F-contrast).

### 2.5 DCM analysis

We applied DCM12.5 (7479) in SPM12 (v7771) to investigate interhemispheric connectivity between OFA and FFA regions for different stimulus conditions. In the following, we describe the definition of model space and the assessment of model parameters.

#### 2.5.1 Model space

Our models included six regions: the left and right V1, the left and right OFA, and the left and right FFA. The bilateral V1 was included in the network model as we had clear assumptions about how visual input drives the system for both central and peripheral stimulation (i.e., the choice of the C-matrix was undisputed). The bilateral OFA and FFA were included because they were key components of the core system of face perception (Haxby et al., 2000,^2002^). Similar network models were also used in other DCM studies concerning the selection of regions (e.g.,Chen et al., 2008;Frässle et al., 2016a,2016b,2016c;Lee et al., 2022). Of course, many more regions are involved in the processing of faces (Duchaine & Yovel, 2015). It would have been possible, for example, to include further regions of the core system (e.g., the pSTS;He et al., 2015;He & Johnson, 2018;Kessler et al., 2021), to differentiate the FFA into different subregions (e.g., an anterior and posterior cluster;Weiner & Grill-Spector, 2012), or to include regions of the extended system (Lohse et al., 2016;Thome et al., 2021). However, we decided against this approach for several reasons. First, larger models may be less robust for the estimation of network parameters (Litvak et al., 2019). Second, it is in our experience possible to determine the location of the bilateral OFA and FFA in most subjects accurately. In contrast, it is more difficult to determine for instance clusters associated with the posterior and anterior FFA in most subjects. Third, and most importantly, we found that for the specific question, that is, the comparison of interhemispheric network connectivity for central and peripheral stimulation, more complex models regarding further network nodes did not seem necessary. The purpose of model selection is ultimately to determine, from a range of plausible alternatives, the most useful model, representing the best balance between accuracy and complexity (Stephan et al., 2010).

We constructed five different models (M1–M5). All models were linear, single state and non-stochastic. Endogenous connectivity (i.e., A-matrix) and exogenous inputs (i.e., C-matrix) were identical for all models. They differed only in their modulatory input (i.e., B-matrix).

The intrinsic structure (A-matrix) included forward connections from V1 to ipsilateral OFA and FFA, ipsilateral, bidirectional connections between OFA and FFA, as well as interhemispheric connections between OFAs and FFAs. Additionally, as implemented by default in DCM, all regions included self-connections. We deliberately avoided a hierarchy between OFA and FFA, as various studies now question such a strict hierarchy (Rossion, 2022). Within each hemisphere, both regions interact, therefore, bidirectionally with each other and both receive equal input from the ipsilateral V1. Between the hemispheres, we assumed homologous connections between the OFAs and the FFAs, but not heterotopic connections between, for example, the left OFA and the right FFA. Diffusion tensor imaging (DTI) studies have shown that these connections, if they exist, are significantly weaker (Catani & Thiebaut de Schotten, 2008;Hofer & Frahm, 2006). We also did not consider interhemispheric connections between bilateral V1. Both postmortem and DTI studies showed that in humans, callosal projections are restricted to higher-order regions, while the V1 is considered to be acallosal (Catani & Thiebaut de Schotten, 2008;Clarke & Miklossy, 1990;Essen et al., 1982;Zilles & Clarke, 1997).

To model external manipulations in the B- and C-matrix, we created a new SPM design matrix with the regressors central stimulation (CS), left stimulation (LS), and right stimulation (RS) (modelling both face and object trials) and the regressors central faces (CF), left faces (LF), and right faces (RF) (modelling the face trials only). C-matrix: All models contained driving inputs to both V1 regions, whereby CS drove both hemispheres, while LS and RS drove only the contralateral region. B-matrix: Connections were modulated by the face regressors CF, LF, and RF. We created five models of increasing complexity due to the higher number of modulatory parameters. In model M1, we only included modulations of the interhemispheric connections. Model M2 had additional modulation on the OFA -> FFA connections, M3 also on the FFA -> OFA connections, M4 on the V1 -> OFA connection, and M5 on the V1 -> FFA connection (seeFig. 2for a graphical illustration).

**Fig. 2.**
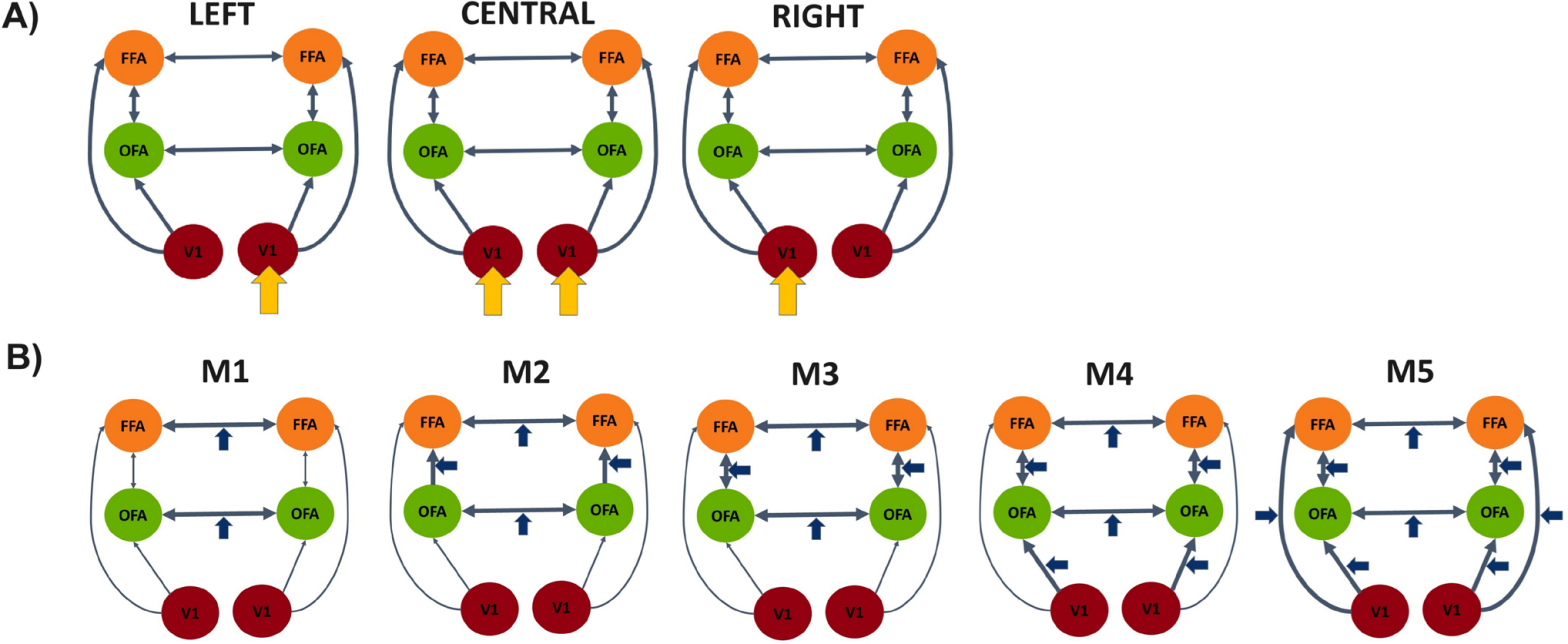
Model space including five models for the DCM analysis. (A) Illustration of endogenous connections (A-matrix, blue arrows) and driving input (C-matrix, yellow arrows) for stimuli (either faces or non-face object) presented centrally or in the left or right hemifield. (B) Illustration of the modulatory influence (B-matrix, large blue arrows) of face stimuli. Models M1–M5 differ in their complexity.

In this study, we did not assume that a specific model would be representative at the population level. We simply believed that some of our chosen models might fit certain individuals better than others. We were, therefore, not interested in inference on the level of model space, but in the model parameters (after model averaging) describing interhemispheric integration (Stephan et al., 2010). Nevertheless, before starting data acquisition, we asked whether the models we had set up were not only plausible but also sufficiently different for DCM to be able to be separated at all. To test this, we calculated simulations in advance. We defined one of the models as the “true” model, generated time series for the individual regions, and used DCM to test whether the true model is reconstructed. Our simulations showed that this worked well and that DCM was able to separate the different models (seeSupplementary Materialpage 9 for details).

#### 2.5.2 Calculation of model parameters

We estimated the model parameters for each model and subject, calculated the model evidence using random-effects Bayesian model selection (BMS) and averaged the parameters across models and subjects using random-effects Bayesian model averaging (BMA) (Zeidman et al., 2019). BMA provides the weighted average of a parameter across all models weighted by the model evidence of each model (Hinne et al., 2020). The A-Matrix hereby represents the effective parameter strength for the baseline. The self-connections represent the self-inhibition of the region where larger values indicate faster decays and smaller values indicate slower decays. The B-Matrix parameters modulate the A-Matrix parameters dependent on the input and can be interpreted as changes to the effective connectivity parameters by either inhibition or excitation. We tested the averaged connection strengths of each parameter for significance based on their posterior probabilities using the spm_Ncfd function that returns the cumulative distributive function with the posterior means and estimated covariances. This function describes the probability for the parameters to be less than zero. All parameters with a posterior probability greater than 0.95 are retrieved for display.

#### 2.5.3 Analysis of the association of stimulus location and interhemispheric transfer

The primary aim of our study was to investigate the influence of stimulus location on interhemispheric transfer as determined by DCM’s interhemispheric modulatory connectivity parameters. We assessed whether a peripheral presentation of the stimulus (i.e., either in the left or right hemifield) leads to an altered interhemispheric transfer between the OFAs and the FFAs compared with a central presentation.

Alterations of the interhemispheric transfer were determined in two ways. On the one hand, we calculated the*interhemispheric transfer difference*(i.e., left-to-right minus right-to-left modulatory connectivity parameter) between the OFAs and the FFAs, respectively. However, we calculated the strength of the interhemispheric transfer (i.e., the average of the absolute value of the left-to-right modulatory connectivity parameter and the absolute value of the right-to-left modulatory connectivity parameter) between the OFAs and the FFAs, respectively. For both measures, we calculated a 2 x 3 factorial ANOVA with the factors region (OFA, FFA) and stimulus location (left, right, central). Our main hypothesis was that the interhemispheric transfer between the OFAs and between the FFAs would be increased for peripheral compared with central stimulus presentation.

## 3 Results

### 3.1 Determination of subject-specific ROIs

Brain activity during face processing was associated with a distributed neural network in the bilateral occipito-temporal cortex as well as frontal and parietal areas. Exemplary activation maps can be taken from theSupplementary Material. At the group level, we specifically found activity in the left FFA, the right FFA, and the right OFA (contrast “F > O”,*p*< 0.001 uncorrected) as well as in the left and right V1 (contrast “F + O > B”,*p*< 0.001 uncorrected) (seeTable 1).

**Table 1.** Center MNI coordinates for the six ROIs included in the DCM analysis.

ROI	Hemisphere	Contrast	X	y	z	Cluster size (voxel)	t-value
OFA	Left		-38	-82	-6	—	—
OFA	Right	F>O	40	-82	-10	3	3.54
FFA	Left	F>O	-40	-44	-24	5	3.82
FFA	Right	F>O	42	-48	-20	50	4.23
V1	Left	F+O>B	-12	-92	-6	239	6.38
V1	Right	F+O>B	16	-88	-2	101	5.65

The coordinates were determined as maxima of a group-level analysis (*p*< 0.001 uncorrected). Bilateral FFA and right OFA were identified for the contrast “F > O,” V1 for “F + O > B.” The left OFA could not be clearly identified at the group level. Therefore, we used coordinates from a previous study (Thome et al., 2022) as a starting point for the determination of the subject-specific coordinates.

Using these group activation maxima as starting point, we determined subject-specific center coordinates for all ROIs included in the DCM analysis (i.e., bilateral V1, OFA, FFA). Since we were not able to locate activation that could be assigned to the left OFA at the group level, we used coordinates from a previous study as a starting point for the determination of the subject-specific coordinates (Thome et al., 2022). Once we had determined the individual local maxima for each ROI and subject, we visually inspected each activation pattern to ensure that these automatically determined coordinates were plausible and represented the activation well. We found that some coordinates were located in regions that did not best match the predefined anatomical areas. We manually adjusted these coordinates to more valid locations by choosing other local maxima. More specifically, we replaced posterior FFA coordinates with more anterior FFA coordinates in four subjects (i.e., sub-09: from [34 -64 -18] to [42 -44 -22]), dorsal OFA values with more ventral coordinates in five subjects (i.e., sub-12: from [-42 -76 -2] to [-32 -82 -12]) and V1 coordinates that were located far-out from the group center coordinates in eight subjects (i.e., sub 18: from [20 -90 -14] to [10 -96 -2]). For two subjects, we had to lower the statistical threshold to determine activation in all ROIs. More specifically, for sub-10, the right OFA was detectable only at*p*= 0.01. For sub-17, the right V1 was detectable at*p*= 0.1. For two subjects, we could not retrieve local maxima for the right OFA (sub-03) or the left OFA (sub-06). These subjects were excluded from further analyses. In the end, the data of 17 participants could be included in the DCM analysis. Overall, the final coordinates for each participant varied mostly for V1 regions (standard deviation across all coordinates: m = 7.09; std = 0.83) and less for OFA (m = 5.33; std = 0.76) and FFA (m = 4.27; std = 2.00). The individual ROI center coordinates are illustrated inFigure 3. A tabular listing (both of the coordinates obtained by SPM’s automated search algorithm and their manual substitutes) is given in the Supplementary Section,Table S4.

**Fig. 3.**
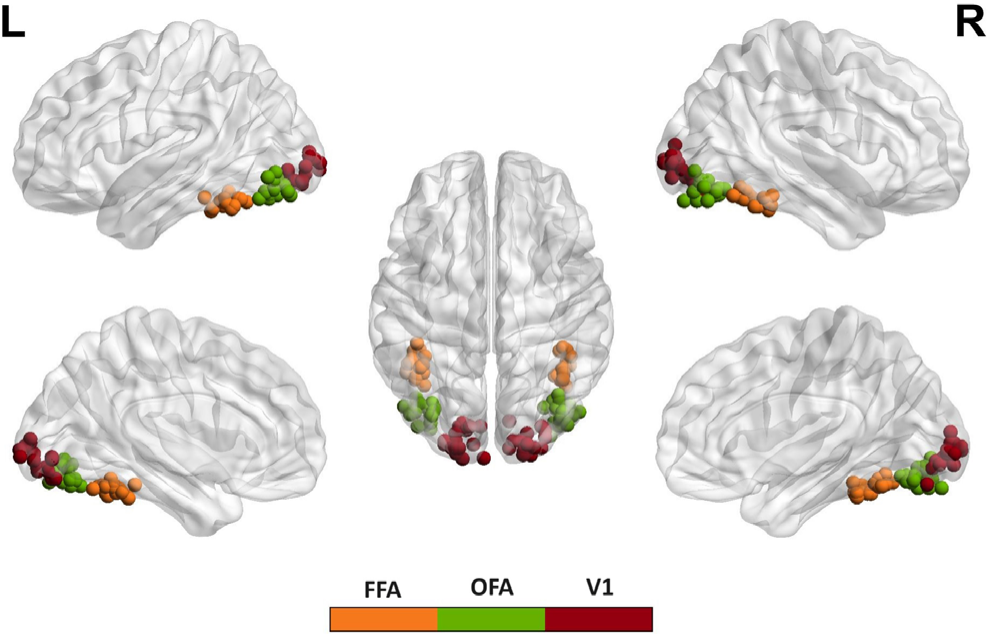
Single subject ROI center coordinates for the six ROIs included in the DCM analysis. The coordinates are presented on an MNI glass brain representation using BrainNet Viewer (Xia et al., 2013).

### 3.2 DCM results

In the following, we (i) present the results of the Bayesian model comparison, (ii) describe the model parameters, and (iii) answer the central question of the study, that is, the association of stimulus location and interhemispheric transfer.

#### 3.2.1 Bayesian model comparison

The models were inverted and then compared using random-effects BMS. We found model M5 to be the most likely model (expected posterior probability 0.47, exceedance probability 0.79; see alsoSupplementary Fig. S2). Model M5 was the most complex model in which the interhemispheric connections between the OFAs and the FFAs as well as all forward connections could be modulated by the face regressors (Fig. 2). After model selection, we used the MATLAB routine spm_dcm_fmri_check to compare the predicted and observed responses over all regions. For model M5, the explained variance was 51.2% averaged across all subjects (std = 13.2%). The model was thus able to reasonably explain the observed data. As a rule of thumb, the explained variance typically should not drop well below 10%, as this indicates a lack of convergence. However, as we also observed large interindividual differences between model preferences using the log Bayes Factors (*log*(*BF*) > 0 for*n*= 9:*M*5 >*M*4;*log*(*BF*) < 0 for*n*= 8:*M*4 >*M*5), we decided to run a random-effects BMA across all models.

#### 3.2.2 Description of model parameters

Random-effects BMA was used to average model parameters across all models and subjects within the pre-specified Occam’s window (*p*< 0.05). Endogenous connectivity parameters (A-matrix) are depicted inFigure 4. We found excitatory forward connections from V1 to the ipsilateral OFA and FFA, whereas all other parameters were inhibitory. Importantly, self-connection parameters (not depicted inFig. 4) were positive, except for the left FFA. Generally, those parameters were approximately four times larger for V1 than for OFA or FFA.

**Fig. 4.**
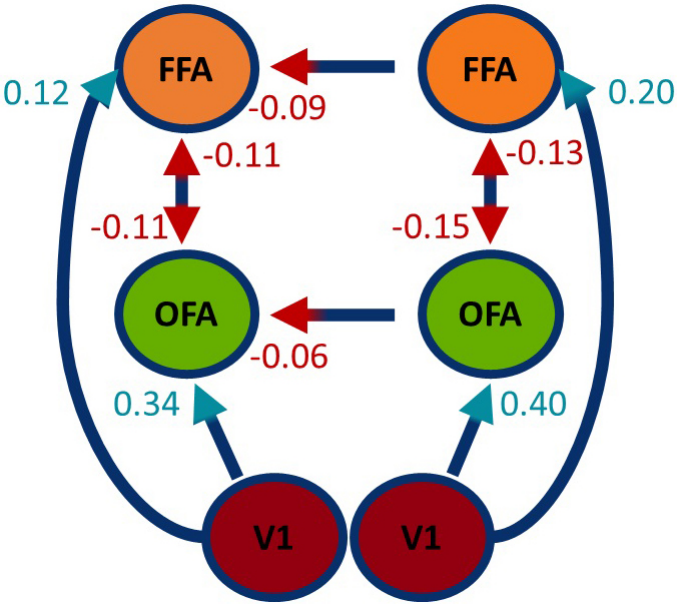
Endogenous connectivity (A-matrix) after BMA across all models and subjects. The strength of connection is shown as the mean of the averaged coupling parameters. Self-connections are omitted in this depiction. Colors indicate the valence of causal influence. Only parameters with a posterior probability > 0.95 are shown.

Modulatory connectivity parameters (B-matrix) and excitatory connectivity parameters (C-matrix) are depicted inFigure 5. In the latter case, direct inputs to V1 were stronger for central than for peripheral presentations (Diff. L = 0.14; Diff. R = 0.20). The modulatory parameters, however, showed greater variability than endogenous connectivity parameters. A detailed list of all parameters is given in the Supplementary Material (Table S5). Presenting the stimulus centrally increased the ipsilateral inputs from V1 to higher-order regions by similar strengths across both hemispheres (for left minus right hemisphere: V1→OFA Diff. = 0.25; V1→FFA Diff. = −0.17). In contrast, larger differences between hemispheres exist for left (V1→OFA Diff. = 0.65; V1→FFA Diff. = 0.73) and right (V1→OFA Diff. = 0.69; V1→FFA Diff. = 0.50) stimulation. Thus, unilateral stimulation preferentially increases the ipsilateral pathways, while bilateral input equally distributes modulatory influences.

**Fig. 5.**
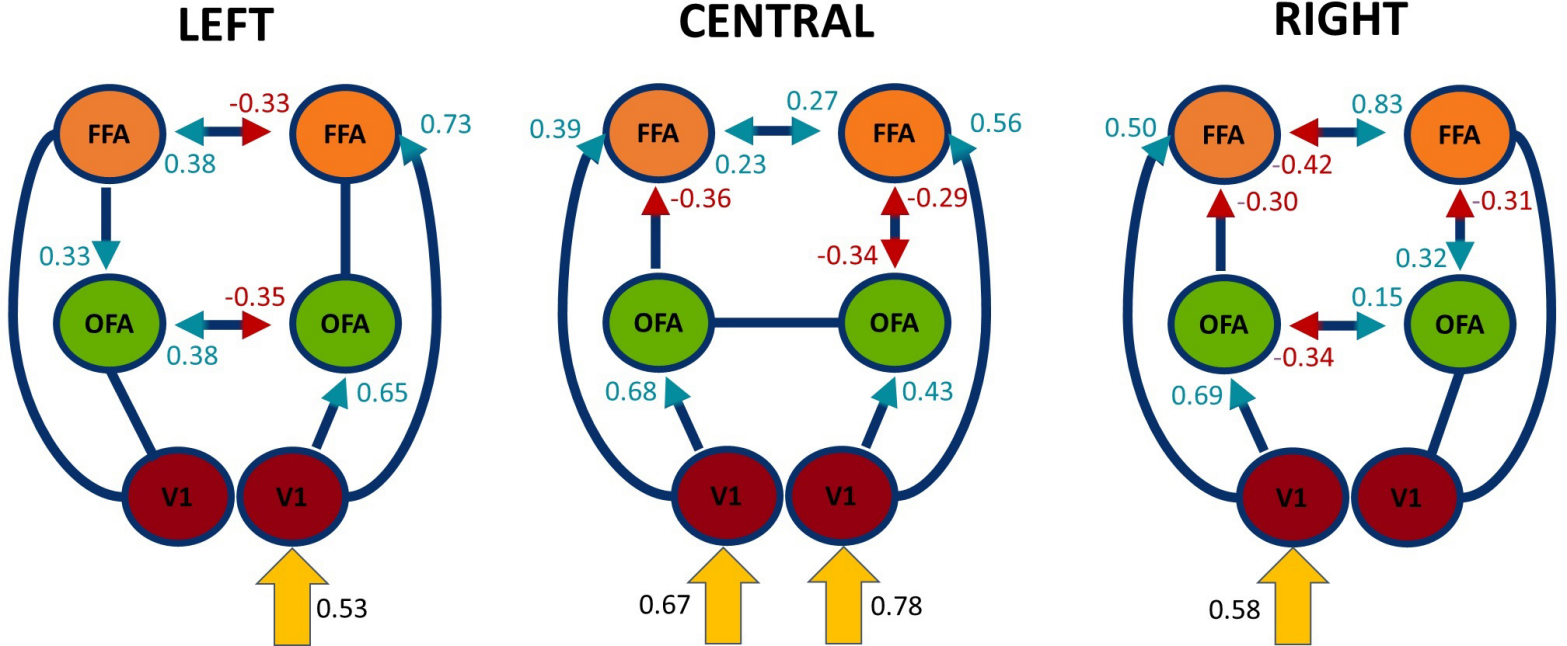
Modulatory (B-matrix) and excitatory (C-matrix) connectivity after BMA across all models and subjects. The strength of connection is shown as the mean of the averaged coupling parameters for LEFT (left side), CENTRAL (middle), and RIGHT (right side) stimulus presentations. Colors (violet, blue) indicate the direction of causal influence of the modulatory parameters. The excitatory parameters are shown in yellow. Only parameters with a posterior probability > 0.95 are shown. Note that the excitatory input enters only one V1 for peripheral stimulation (left / right), but both V1s for central stimulation.

Further, intrahemispheric coupling between OFA and FFA was downregulated by central presentations. This pattern changed for peripheral presentations, where top-down processes from FFA to OFA increased (lFFA→lOFA = 0.33; rFFA→rOFA = 0.32).

Looking at the interhemispheric connectivity parameters, we found increased coupling strength between the FFAs for centrally depicted faces. However, connection strengths between OFAs did not exceed the posterior probability of 0.95. In comparison with central stimulation, peripheral stimulation revealed asymmetrical interhemispheric connectivity patterns for left and right presentations separately. While left presentations (input to right hemisphere) increased coupling strength from right-to-left hemisphere and decreased coupling strength from left-to-right hemisphere, right presentations (input to left hemisphere) decreased the first and increased the second mentioned.

#### 3.2.3 Association of stimulus location and interhemispheric transfer

The primary aim of our study was to investigate the influence of stimulus location (central vs. peripheral) on the interhemispheric transfer in the core system of face perception. The interhemispheric transfer was assessed by DCM’s interhemispheric modulatory connectivity parameters (B-matrix). InFigure 6, we show the strength of both the interhemispheric endogenous and the interhemispheric modulatory connectivity parameters, as calculated by BMA, for left, right, and central stimulus presentation. It is evident that the interhemispheric transfer is mainly determined by the modulatory parameters. On average, the modulatory parameters are nine times stronger than the endogenous parameters. Face-specific interhemispheric connectivity can, therefore, basically be described by the modulatory parameters. InFigure 7A, we additionally present the interhemispheric transfer differences of the modulatory connectivity parameters (i.e., left-to-right MINUS right-to-left modulatory connectivity parameters) for OFAs and FFA. InFigure 7B, we present the average between interhemispheric connectivity (L->R, R->L) calculated as the sum of absolute values divided by 2.

**Fig. 6.**
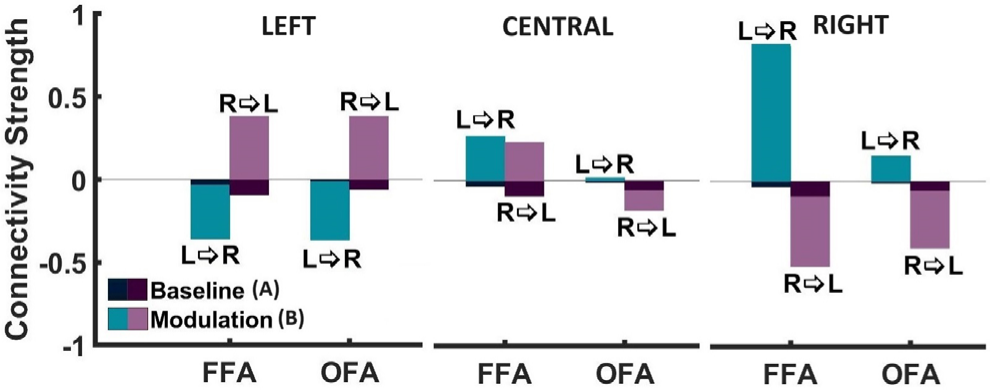
Total interhemispheric connectivity strength. The strength of modulatory, B-Matrix connectivity added to endogenous “Baseline”, A-Matrix connectivity for interhemispheric connectivity between FFAs and OFAs for different stimulus locations. Modulatory effects are the main contributor to connectivity strength changes.

**Fig. 7.**
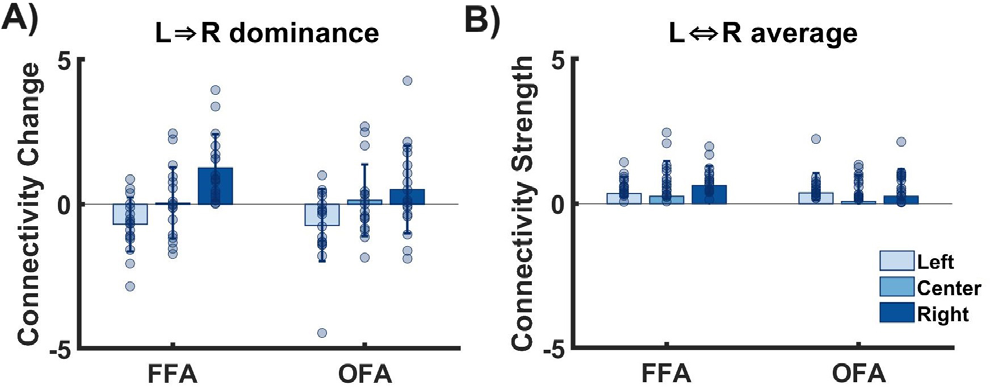
Interhemispheric transfer. (A)Differencebetween interhemispheric modulatory connectivity, calculated as left-to-right minus right-to-left transfer strength, presented for both OFA and FFA as well as different stimuli locations (left, center, right). (B)Averagebetween interhemispheric modulatory connectivity calculated as the sum of absolute values divided by 2.

Descriptively, the central presentation is characterized by a moderately strong excitatory modulatory connectivity between the two FFAs (lFFA -> rFFA = 0.27 ± 0.13, rFFA -> lFFA = 0.23 ± 0.13) and a low excitatory (lOFA -> rOFA = 0.02 ± 0.13) and moderately strong inhibitory connectivity (rOFA -> lOFA = -0.12 ± 0.10) between the two OFAs. The strength of the connectivity between the homologous areas is relatively symmetrical, that is, the interhemispheric transfer difference is low (FFA transfer difference = 0.04 ± 1.24, OFA transfer difference = 0.14 ± 1.24). In contrast, the peripheral presentation is characterized by a fundamentally different connectivity pattern. For both the OFA and the FFA connections, the modulatory connectivity from the hemisphere receiving the visual input to the opposite hemisphere is strongly excitatory, while the modulatory connectivity in the opposite direction is strongly inhibitory. For example, if the stimulus is presented in the left visual field, the input enters the primary visual cortex of the right hemisphere. The transfer from right to left is positive, while the transfer from left to right is negative. Regarding the strength of the individual connectivity parameters, it is noticeable that they tend to be stronger for peripheral stimulation than for central stimulation.

A formal statistical analysis (2 x 3 factorial ANOVA) of theinterhemispheric transfer difference(i.e., left-to-right MINUS right-to-left modulatory connectivity parameter, seeFig. 7A) revealed a significant main effect of stimulus location (*F*= 14.23,*df*= 2,*p*< 0.001). Post hoc tests showed that the transfer differences were significantly different between all three stimulus locations both for the OFA connection and for the FFA connection. Peripheral stimulation was associated with increased interhemispheric transfer differences compared with central stimulation at the level of both the OFA and the FFA, confirming our main hypothesis. We did not find a significant main effect of region (*F*= 0.87,*df*= 1,*p*= 0.353) nor a significant interaction between stimulus location and region (*F*= 1.19,*df*= 2,*p*= 0.309). This showed that there were no recognizable differences between the OFA and the FFA connections.

A statistical analysis of theinterhemispheric transfer strength(i.e., the average of the absolute value of the left-to-right modulatory connectivity parameter and the absolute value of the right-to-left modulatory connectivity parameter, seeFig. 7B) revealed no significant main effect of stimulus location (*F*= 1.04,*df*= 2,*p*= 0.359), no significant main effect of region (*F*= 1.8,*df*= 1,*p*= 0.183), and no significant interaction between stimulus location and region (*F*= 0.57,*df*= 2,*p*= 0.568). A change in interhemispheric connectivity was, therefore, rather implemented by changes in left-right and right-left connectivity in opposite directions, not by a general increase in connectivity strength.

## 4 Discussion

In this study, we used DCM to examine whether the location of stimulus presentation (central vs. peripheral) affects interhemispheric network connectivity within the core system of face perception. Our findings revealed a significantly greater interhemispheric transfer difference between the bilateral OFAs and FFAs for peripheral (i.e., left or right) compared with central presentations, supporting our main hypothesis. However, we did not observe a general increase in interhemispheric transfer strength. In the following sections, we will discuss the results of the study, with a particular focus on their implications for investigating the left- and right-hemispheric homologues of the face network (4.1). We will then outline the study’s limitations (4.2) before concluding with a short summary (4.3.).

### 4.1 Differences of the face network for peripheral compared with central presentations

The face processing network is inherently bilateral, with both the OFA and FFA being present in the left and right hemispheres. However, the functional differences between these homologous regions remain incompletely understood (Meng et al., 2012), as do the factors driving individual differences in lateralization (Thome et al., 2022) and the mechanisms underlying interhemispheric interactions. Despite this, neuroimaging studies on face processing often focus exclusively on the right hemisphere, overlooking the bilateral nature of these regions (Fairhall & Ishai, 2007;Li et al., 2010;Nguyen et al., 2014;Tsantani et al., 2021).

To address this gap, we systematically investigated interhemispheric connectivity in the face processing network across multiple studies. Using fMRI and DCM, we examined the network properties of face perception, with a particular focus on interhemispheric interactions between the left and right OFA and between the left and right FFA (Frässle et al., 2016a,2016b,2016c). These studies employed peripheral stimulus presentation, enabling the exploration of interhemispheric processing mechanisms, for instance, to distinguish between interhemispheric inhibition and recruitment (for an overview of analytical principles, seeStephan, Fink, et al., 2007).

This approach yielded highly reliable results both within subjects and across different subject samples (Frässle et al., 2016b). It provided valuable insights into the mechanisms underlying hemispheric lateralization (Frässle et al., 2016a), enabled a mechanistic understanding of lateralization differences between right- and left-handers (Frässle et al., 2016c), and demonstrated that specific network connectivity parameters—particularly interhemispheric transfer differences—were linked to both structural (Frässle et al., 2016a) and physiological markers, such as pupil responses (Frässle et al., 2016a,2016b).

However, it is important to recognize that the specific experimental design can significantly influence connectivity patterns. In particular, peripheral stimulus presentation can substantially modulate interhemispheric interactions. As a result, the neural network parameters derived from a DCM analysis may differ between peripheral and foveal stimulus presentations. In the present study, we directly compared the effects of central and peripheral stimulation on network connectivity to better understand these differences.

Our present results demonstrate that the location of the stimulus has a significant impact on interhemispheric connectivity and the overall connectivity pattern. For central presentations, the face processing network exhibits moderately strong excitatory connectivity between the two FFAs, coupled with moderately strong inhibitory connectivity between the two OFAs (albeit the latter was not significant). The strength of connectivity between the homologous regions is symmetrical, resulting in a low interhemispheric transfer difference. In contrast, for peripheral presentations, the connectivity pattern shifts fundamentally. Both the interhemispheric connections between the OFA and FFA are characterized by excitatory connectivity from the hemisphere receiving the visual input to the opposite hemisphere, while the reverse direction shows inhibitory modulation. For instance, when the stimulus is presented in the left visual field, the input is processed in the primary visual cortex of the right hemisphere. The transfer from the right to the left hemisphere is positive, whereas the transfer from the left to the right hemisphere is negative.

When comparing network connectivity during central and peripheral stimulation, the pattern observed during peripheral stimulation may reflect compensatory mechanisms. These compensation processes likely involve changes in interhemispheric connections, with positive transfer to the respective homologous regions of the FFA and OFA. Such mechanisms may aim to rebalance network dynamics towards the preferred state of bilateral representation. Interestingly, interhemispheric transfer differs in the interhemispheric transfer difference but not in the interhemispheric transfer strength, suggesting that peripheral stimulation does not require more resources than central stimulation.

While direct interhemispheric exchange between homologous V1 regions is not modelled, given the lack of substantial callosal connections at this level, the onset and progression of significant interhemispheric transfer within the face processing network remain a topic of ongoing debate. We observed positive interhemispheric transfer between bilateral FFAs, suggesting significant interactions at this level. By contrast, interhemispheric transfer between the OFAs was inhibitory and non-significant. A first look at the results (e.g.,Fig. 5) might suggest that this increasing trend is less pronounced or absent during peripheral stimulation. However, we found no significant difference in overall interhemispheric transfer strength between central and peripheral stimulation. Future studies specifically targeting interhemispheric transfer at distinct processing stages are needed to fully elucidate its evolution within the face processing system.

Interestingly, we observed a difference in interhemispheric transfer between left visual field stimulation and central stimulation, despite face information entering the dominant right hemisphere in both cases. While central stimulation resulted in balanced transfer between homologous regions, left visual field stimulation led to relatively greater right-to-left transfer, with the dominant right hemisphere even experiencing inhibitory influence from the left. Thus, even when input is restricted to the right hemisphere, the left hemisphere is recruited. This challenges studies that limit investigations of the face perception system to the right hemisphere, as the left hemisphere likely plays a role beyond the functions of its dominant right homologue.

Although our study was not designed to directly address the underlying connectivity mechanisms of right lateralization in face processing (see, e.g.,Frässle et al., 2016c), we can still offer some interesting descriptive observations. During right visual field stimulation, we observed strong interhemispheric transfer at the FFA level (i.e., significant transfer from the left FFA to the right FFA). This suggests substantial information flow to the dominant right hemisphere when face information initially enters the non-dominant left hemisphere. In contrast, interhemispheric transfer between the FFAs appears balanced during central stimulus presentation. Thus, right-lateralization during central stimulation is more likely explained by intrahemispheric transfer from right V1 to right FFA. Similarly, we observed strong intrahemispheric transfer from right V1 to right FFA during left visual field stimulation. In summary, while right visual field stimulation may induce right-lateralized FFA activity through interhemispheric transfer, right lateralization during central and left visual field presentations is more likely driven by intrahemispheric mechanisms.

At this point, we would like to briefly address the lateralization of brain activation observed in this study. In a previous fMRI study, we reported that fMRI activity during a standard face processing task (a typical block design “face localizer”) was, on average, stronger in right-hemispheric regions than in their left-hemispheric counterparts. However, this asymmetry was relatively subtle compared with other lateralized brain functions, such as language and spatial attention, and exhibited substantial individual variability (Thome et al., 2022). When applying the same criteria commonly used in language lateralization studies, only half of the participants in that study could be classified as right-hemispheric dominant. Based on these findings, we concluded that the lateralization of the face perception network, as measured with fMRI, is not strongly right dominant—at least not when considering individual differences.

At first glance, our previous findings may seem inconsistent with the current results, as the present study shows a clear right-hemispheric dominance in brain activation at the group level (seeTable 1). However, this apparent discrepancy disappears when individual lateralization patterns are taken into account. In theSupplementary Table S7we provide the lateralization index (LI) for V1, OFA, and FFA under foveal stimulus presentation, where the substantial variability between participants becomes evident. For example, when using the “negative LI” cutoff criterion, only 14 out of 20 participants can be classified as right dominant in the FFA. If a more stringent threshold for right-hemispheric dominance is applied (e.g., LI lower than -0.2, a criterion commonly used in language lateralization studies), this number decreases further, with only 8 out of 20 participants classified as “clearly right-dominant.”

A noteworthy factor contributing to the substantial differences between group-level and individual analyses is likely the variability in the anatomical location of face-processing regions between the left and right hemispheres. For example,Rossion et al. (2012)report that the localization of the left FFA varies significantly more across individuals than that of the right FFA. This observation aligns with findings from the dataset ofThome et al. (2021). As a consequence, left-hemispheric face regions may appear less activated in classical univariate group analyses, simply due to greater interindividual variability in their precise anatomical positioning.

### 4.2 Limitations of the study

The long-term goal of our studies is to gain a deeper understanding of the functional differences between the left- and right-hemispheric homologues of the face processing network. The combination of neuroimaging and network analyses, together with peripheral visual stimuli, may offer a promising approach to further investigate these differences. However, it is important to acknowledge that the design we have chosen carries certain inherent limitations. These limitations are not critical for the aims of the present study, which primarily sought to explore how network parameters derived from DCM might differ between central and peripheral stimulation. Nonetheless, it is crucial to be aware of these limitations when planning future studies and interpreting their results. Therefore, we would like to discuss these limitations in detail in the following.

The first limitation arises from the fact that peripheral and central face stimuli differ not only in their location of presentation, and thus in the initial activation of the early visual cortex (left vs. right V1), but also lead to a distinctly altered processing strategy, which could introduce additional differences at the neural level. For the interpretation of the results, it is, therefore, important to carefully consider how to differentiate between these two factors—the different stimulus location and the resulting differences in processing.

More specifically, the paradigm required participants to fixate on the presented stimuli and, as an additional task, they were instructed to press a button on a response box whenever a cross-hair accompanying the stimuli turned red. However, this additional task did not necessitate a deeper analysis of the presented stimuli, potentially leading to a more “shallow” processing style. This may have been particularly pronounced for the peripherally presented stimuli, which were more challenging to perceive. For example, when stimuli are presented in the periphery, they can be categorized as faces but typically not individualized very well (at least for the short 150 ms presentation) as compared with a foveal presentation. This might lead to a weaker fMRI for faces presented in the periphery, not least because of adaptation for faces in the periphery (which looks similar) compared with central presentation. Therefore, it would be valuable to explore whether the connectivity pattern is influenced by the specific design of the face-processing paradigm. For instance, investigating whether a longer presentation time or additional judgements on the stimuli (e.g., symmetry or sex) could alter this pattern would be an interesting direction for future research. One way to control for the increased signal adaptation in the periphery would also be to use a design in which the same face identity is repeated during a block.

It is also important to consider the extent to which the results depend on the chosen visual angle. In a pilot study conducted prior to this experiment, we presented the stimuli with two different angles—once at 4.02° and once further in the periphery. We observed that the farther the stimuli were presented from the center, the lower the activation was in V1, OFA, and FFA. Based on these findings, we selected a visual angle of 4.02°, as it provided a balance between parafoveal stimulation and sufficiently strong activation. While we expect that brain activation, and potentially hemispheric lateralization, may vary with different visual angles, we still anticipate that any form of peripheral stimulation will lead to changes in the connectivity pattern compared with central presentation.

A second limitation relates to the visual field representations of the OFA and FFA. fMRI studies of population receptive fields indicate a contralateral bias in face-sensitive regions within the core face perception network (Finzi et al., 2021). Importantly, these regions do not contain complete representations of the visual field, with sensitivity declining beyond 15–20° of eccentricity. Moreover, there are distinct eccentricity biases for instance between the OFA and FFA or between subregions of the FFA (Finzi et al., 2021;Poltoratski et al., 2021), which may influence how these regions process peripheral stimuli. For instance, if the FFA ROI for some participants primarily captures activity in the more anterior portion of the fusiform gyrus (mFus-faces), while in others it predominantly reflects activation in the posterior cluster (pFus-faces), this introduces a potential bias that complicates comparisons across subjects. Notably, receptive field information was not incorporated into the neural models used in the current study.

A third key limitation concerns the temporal characteristics of the receptive fields of the OFA and FFA. The receptive fields are unlikely to be static. Instead, they may initially show a strongly lateralized representation, followed by a more bilateral pattern. Assessing these dynamic changes is typically beyond the temporal resolution of fMRI, as the temporal response of neurons is much faster than the fMRI response (but seeKim et al., 2024, for a framework to estimate spatiotemporal receptive fields from fMRI data). In future studies, intracranial human recordings could be employed to also investigate the temporal changes in representations and to examine how these affect the network connectivity (seeMcCarthy et al., 1999, for lateralized presentations of faces in intracranial recordings).

In future studies, further aspects that remain unresolved in the present study could be explored. Here, we specifically examined whether the location of stimulus presentation influences the face processing network. An interesting avenue for further research would be to explore whether similar changes in interhemispheric connectivity occur during the processing of non-face stimuli, such as scenes or bodies. Another important question is whether the observed connectivity modulations within the network are due to the lateralized nature of stimulation or simply by the fact that the stimuli are presented far from the fovea. To address this, future studies could present face stimuli for instance in the upper and lower visual field at an eccentricity of 4.02° from the central fixation point and assess potential changes in the connectivity pattern.

### 4.3 Conclusion

In conclusion, the characteristics of the face processing network, as derived from DCM analysis, are notably influenced by the location of the stimulus and exhibit differences between peripheral and foveal presentations. While peripheral stimulation can be a useful tool for investigating specific properties of the face network in carefully designed experiments, it does not provide network parameters that are representative of the more typical, foveal processing of faces. The mechanisms underlying interhemispheric integration for peripheral stimuli appear to differ from those involved in central stimulation. Therefore, caution should be exercised when attempting to generalize findings from peripheral face processing to face processing more broadly.

## Supplementary Material

Supplementary Material

## Data Availability

The codes and preprocessed, defaced data are made available at the OSF website under the following link:https://doi.org/10.17605/OSF.IO/T9YM6
