# Supplementary material for: Interhemispheric integration in the neural face perception network: Does stimulus location matter?
Source: Imaging Neurosci (Camb). 2025 May 29;3:IMAG.a.17. doi: 10.1162/IMAG.a.17 (PMC12320007; doi:10.1162/IMAG.a.17)
Supplement: Supplementary Material [file imag.a.17_supp.pdf]

Supplementary Material for the study on “Interhemispheric Integration in the Neural Face Recognition Network: Does Stimulus Location Matter?”

Julia Elina Stocker [1], Antonia Schulz [1], Ina Thome [1], Jens Sommer [1], Jonas Rabeneck [1], Kristin Rusch [1], Olaf Steinsträter [1], Andreas Jansen [1]

This supplement contains additional reports on the data from the FNI (Face Network Integration) study. The study was preregistered on OSF under https://doi.org/10.17605/OSF.IO/T9YM6 where preprocessed fMRI images of the study participants and the pilot data can be retrieved. The codes used for analysis are published in the same repository. For further information please contact the corresponding author:

Julia Elina Stocker, Department of Psychiatry and Psychotherapy, Philipps University of Marburg, Germany

Rudolf-Bultmann-Straße 8, D-35039 Marburg

E-Mail: elina.stocker@uni-marburg.de

Table of Contents

Demographics ... 2
MRIQC ... 3
ROI Analysis ... 3
Bayesian Model Comparison ... 9
BMS Validation ... 10
Parameter Averaging ... 11
Piloting ... 13
Resources ... 14

Demographics

Table 1: Participant Information

ID	Sex	Age	Handedness Scores	Handed-ness	Vision Correction	Colour-blindness	Bilingual	Reaction Times (Mean ± Std)	Accuracy (%)
01	f	21	80	R	contact lenses	0	0	0.36 ± 0.06	98.96
02	m	22	67	R	glasses	0	0	0.41 ± 0.13	100.00
03	m	33	73	R	contact lenses	0	1	0.50 ± 0.11	95.83
04	m	25	100	R	-	0	1	0.38 ±0.07	96.88
05	f	25	100	R	contact lenses	0	0	0.49 ± 0.13	96.88
06	f	23	41	R	glasses	0	0	0.36 ±0.04	100.00
07	m	23	88	R	-	1	0	0.43 ±0.09	97.92
08	m	30	82	R	-	0	1	0.37 ±0.06	100.00
09	f	23	38	R	glasses	0	0	0.33 ±0.05	100.00
10	f	28	100	R	glasses	0	0	0.36 ±0.05	97.92
11	f	24	88	R	glasses	0	0	0.41 ±0.07	98.96
12	m	29	100	R	-	0	0	0.38 ±0.05	98.96
13	m	23	60	R	-	0	0	0.38 ±0.07	98.96
14	f	18	100	R	-	0	0	0.42 ±0.11	95.83
15	x	21	100	R	contact lenses	0	0	0.44 ±0.12	89.58
16	f	40	100	R	glasses	0	0	0.38 ±0.06	98.96
17	f	36	100	R	-	0	0	0.36 ±0.08	96.88
18	f	21	100	R	-	0	0	0.37 ±0.05	94.79
19	m	28	100	R	glasses	0	0	0.39 ±0.07	98.96
20	m	21	100	R	-	0	0	0.43 ±0.08	96.88

Note. Participant table with demographic information as well as reaction times and accuracy for the attention task.

MRIQC

Quality assessment of MRI images is an important procedure to ensure reliability of the results. Unfortunately, there are many ways on how to perform quality control on the data. From simple visual inspection to a multitude of different control measures, there is a large variety that has been adapted by labs all over the world, leading to a high variability and less comparability between data sets (Esteban et al., 2017). A standardized pipeline, that tries to counteract this issue is MRIQC. MRIQC performs quality control of structural and functional MRI data. It is used to create image quality metrices (IQMs) of the data provided and allows the user to rate these under visual inspection. These IQMs indicate the amount of noise, the spatial distribution of information in the data, the impact of artifacts, tissue distributions, volume overlaps, image blurriness and more. As these numbers are difficult to interpret we used mriqception (*mriqception.* (2019). Github: Neurohackademy. Retrieved from https://github.com/ elizabethbeard/mriqception) to compare our study data to a database of over 200 000 images.

Table 2: Image quality metrices

IQM		FNI Data (20 participants)		MRIQC Database (>200 000 images)	
EFC		0.56	[0.54; 0.58]	0.47	[0.44;0.53]
FBER		949.06	[767.98;1086.70]	2404.84	[928.62;17409.88]
FWHM (mm)	x	2.23	[2.18;2.26]	2.54	[2.29;2.70]
	y	2.52	[2.47;2.57]	2.95	[2.69;3.24]
	z	2.02	[1.96;2.06]	2.33	[2.24;2.46]
GSR	x	-0.01	[-0.01;0.00]	-0.01	[-0.01;0.006]
GSR	y	0.05	[0.04;0.05]	0.02	[0.01;0.03]
SNR		4.57	[4.38;4.65]	4.52	[4.16 ;5.25]
DVARS std		1.08	[1.06;1.10]	1.19	[1.13;1.27]
FD (mm) mean		0.17	[0.14;0.19]	0.20	[0.12;0.30]
GCOR		0.03	[0.02;0.05]	0.03	[0.02;0.06]
TSNR		48.44	[44.80;52.77]	55.06	[44.06;63.52]
AOR		0.00	[0.00;0.00]	0.003	[0.002;0.005]
AQI		0.01	[0.01;0.01]	0.007	[0.005;0.01]

Note. Quality metrices of our data and the standard MRIQC Database.

ROI Analysis

Table 3: Individual ROI Coordinates

ID	ROI	Hemisphere	Contrast	x	y	z	Cluster size (voxel)	t-value
01	FFA	Left	F>O	-42	-38	-20	45	6,42
01	FFA	Right	F>O	36	-56	-20	83	Inf
01	OFA	Left	F>O	-32	-74	-22	63	7,02
01	OFA	Right	F>O	50	-76	-20	2	3,53
01	V1	Left	F+O>B	-26	-92	-12	123	Inf
01	V1	Right	F+O>B	18	-100	2	118	Inf
04	FFA	Left	F>O	-38	-54	-20	45	Inf
04	FFA	Right	F>O	38	-54	-20	113	Inf
04	OFA	Left	F>O	-50	-72	-8	50	Inf
04	OFA	Right	F>O	44	-84	-4	61	7,6
04	V1	Left	F+O>B	-18	-96	6	123	Inf
04	V1	Right	F+O>B	18	-94	14	123	Inf
05	FFA	Left	F>O	-40	-48	-24	110	Inf
05	FFA	Right	F>O	40	-56	-20	122	Inf
05	OFA	Left	F>O	-34	-78	-18	122	Inf
05	OFA	Right	F>O	38	-72	-18	113	Inf
05	V1	Left	F+O>B	-20	-88	-10	123	Inf
05	V1	Right	F+O>B	32	-82	-12	123	Inf
07	FFA	Left	F>O	-48	-54	-24	60	7,42
07	FFA	Right	F>O	40	-54	-16	110	Inf
07	OFA	Left	F>O	-42	-68	-20	119	12,86
07	OFA	Right	F>O	28	-72	-16	94	Inf
07	V1	Left	F+O>B	-14	-86	-12	123	Inf
07	V1	Right	F+O>B	18	-98	2	123	Inf
08	FFA	Left	F>O	-44	-46	-22	83	Inf
08	FFA	Right	F>O	42	-42	-20	48	7,51
08	OFA	Left	F>O	-46	-78	-6	78	Inf
08	OFA	Right	F>O	40	-76	-10	94	Inf
08	V1	Left	F+O>B	-22	-100	2	93	Inf
08	V1	Right	F+O>B	34	-90	-6	123	Inf
09	FFA	Left	F>O	-44	-42	-26	88	7,37
09	FFA	Right	F>O	42	-44	-22	93	9,07
09	OFA	Left	F>O	-44	-68	-16	95	Inf
09	OFA	Right	F>O	38	-80	-8	48	6,74
09	V1	Left	F+O>B	-32	-94	-2	123	Inf
09	V1	Right	F+O>B	28	-98	2	119	Inf
10	FFA	Left	F>O	-36	-62	-22	27	5,08
10	FFA	Right	F>O	40	-42	-26	14	3,64
10	OFA	Left	F>O	-42	-84	-16	2	3,35
10	OFA	Right	F>O	40	-78	-10	1	2,49
10	V1	Left	F+O>B	-16	-102	0	123	Inf
10	V1	Right	F+O>B	26	-98	-4	123	18,59
11	FFA	Left	F>O	-38	-44	-24	60	6,03
11	FFA	Right	F>O	38	-42	-24	59	6,38
11	OFA	Left	F>O	-32	-70	-18	74	Inf
11	OFA	Right	F>O	32	-90	-20	1	3,15
11	V1	Left	F+O>B	-4	-102	-4	113	15,39
11	V1	Right	F+O>B	12	-92	0	118	14,09
12	FFA	Left	F>O	-46	-50	-22	56	6,53
12	FFA	Right	F>O	38	-58	-18	92	Inf
12	OFA	Left	F>O	-32	-82	-12	23	5,08
12	OFA	Right	F>O	38	-84	-16	112	Inf
12	V1	Left	F+O>B	-6	-84	-6	96	7,05
12	V1	Right	F+O>B	20	-94	4	123	Inf
13	FFA	Left	F>O	-40	-52	-16	76	Inf
13	FFA	Right	F>O	42	-48	-24	123	Inf
13	OFA	Left	F>O	-32	-76	-10	94	Inf
13	OFA	Right	F>O	44	-72	-16	85	Inf

13	V1	Left	F+O>B	-18	-84	-18	123	Inf
13	V1	Right	F+O>B	20	-94	12	123	Inf
14	FFA	Left	F>O	-40	-42	-28	52	5,51
14	FFA	Right	F>O	44	-50	-24	110	Inf
14	OFA	Left	F>O	-42	-76	-14	46	7,04
14	OFA	Right	F>O	36	-66	-16	63	6,3
14	V1	Left	F+O>B	-20	-96	6	123	Inf
14	V1	Right	F+O>B	20	-88	-8	121	Inf
15	FFA	Left	F>O	-38	-50	-20	48	6,44
15	FFA	Right	F>O	40	-58	-14	85	Inf
15	OFA	Left	F>O	-36	-72	-18	103	Inf
15	OFA	Right	F>O	38	-66	-14	65	Inf
15	V1	Left	F+O>B	-20	-92	-12	118	Inf
15	V1	Right	F+O>B	30	-86	-6	123	Inf
16	FFA	Left	F>O	-48	-52	-26	38	5,9
16	FFA	Right	F>O	40	-40	-18	114	Inf
16	OFA	Left	F>O	-40	-72	-16	98	Inf
16	OFA	Right	F>O	38	-70	-14	99	Inf
16	V1	Left	F+O>B	-20	-80	-16	123	Inf
16	V1	Right	F+O>B	18	-98	-8	114	11,43
17	FFA	Left	F>O	-42	-56	-20	102	Inf
17	FFA	Right	F>O	42	-62	-18	116	Inf
17	OFA	Left	F>O	-34	-78	-8	18	5,58
17	OFA	Right	F>O	36	-74	-14	120	Inf
17	V1	Left	F+O>B	-10	-94	-6	122	Inf
17	V1	Right	F+O>B	10	-80	-20	119	Inf
18	FFA	Left	F>O	-46	-48	-20	82	7,45
18	FFA	Right	F>O	40	-40	-24	72	Inf
18	OFA	Left	F>O	-32	-72	-16	48	5,46
18	OFA	Right	F>O	46	-78	-14	74	7,11
18	V1	Left	F+O>B	-12	-84	-4	123	12,87
18	V1	Right	F+O>B	10	-96	-2	121	10,63
19	FFA	Left	F>O	-42	-58	-20	108	Inf
19	FFA	Right	F>O	40	-52	-18	96	Inf
19	OFA	Left	F>O	-44	-76	-10	105	Inf
19	OFA	Right	F>O	38	-80	-12	70	7,24
19	V1	Left	F+O>B	-20	-92	-10	123	Inf
19	V1	Right	F+O>B	16	-92	-2	123	Inf
20	FFA	Left	F>O	-38	-50	-20	28	6,24
20	FFA	Right	F>O	44	-44	-20	33	6,51
20	OFA	Left	F>O	-34	-84	-18	67	6,67
20	OFA	Right	F>O	34	-82	-22	63	5,97
20	V1	Left	F+O>B	-24	-96	4	123	Inf
20	V1	Right	F+O>B	24	-96	4	119	Inf

**Summary Statistics**			x	y	z	
			Mean ± Std			
FFA	Left	F>O	-41,76 ± 3,73	-49,76 ± 6,20	-22,00 ± 3,00	65,18
FFA	Right	F>O	40,35 ± 2,15	-49,53 ± 7,26	-20,35 ± 3,26	87,24
OFA	Left	F>O	-38,12 ± 5,94	-75,29 ± 5,00	-14,47 ± 4,67	70,88
OFA	Right	F>O	38,71 ± 5,24	-76,47 ± 6,54	-14,35 ± 4,60	68,53
V1	Left	F+O>B	-17,76 ± 7,03	-91,88 ± 6,65	-5,53 ± 7,47	118,71
V1	Right	F+O>B	20,82 ± 7,25	-92,71 ± 5,83	-1,65 ± 8,31	120,94

Table 4: Coordinate Adjustment

ID	ROI	Hemisphere	old			new			p-value (uncorr.)
			x	y	z	x	y	z	
01	OFA	Right	50	-78	4	50	-76	-20	0.001
07	OFA	Left	-44	-88	4	-42	-68	-20	0.001
08	FFA	Right	46	-60	-16	42	-42	-20	0.001
09	FFA	Right	34	-64	-18	42	-44	-22	0.001
10	OFA	Right	0	0	0	40	-78	-10	0.01
10	V1	Right	26	-86	6	26	-98	-4	0.001
11	FFA	Right	36	-60	-12	38	-42	-24	0.001
11	V1	Left	-28	-86	-14	-4	-102	-4	0.001
11	V1	Right	26	-84	-14	12	-92	0	0.001
12	OFA	Left	-42	-76	-2	-32	-82	-12	0.001
12	V1	Left	-26	-86	-12	-6	-84	-6	0.001
16	V1	Right	22	-92	16	18	-98	-8	0.001
17	V1	Right	28	-78	-6	10	-80	-20	0.1
18	V1	Left	-22	-100	6	-12	-84	-4	0.001
18	V1	Right	20	-90	-14	10	-96	-2	0.001
20	FFA	Right	48	-60	-18	44	-44	-20	0.001
20	OFA	Right	44	-76	-12	34	-82	-22	0.001

Note. Change in coordinates to more appropriate maxima.

Figure 1: Activation maps for F>O Contrast

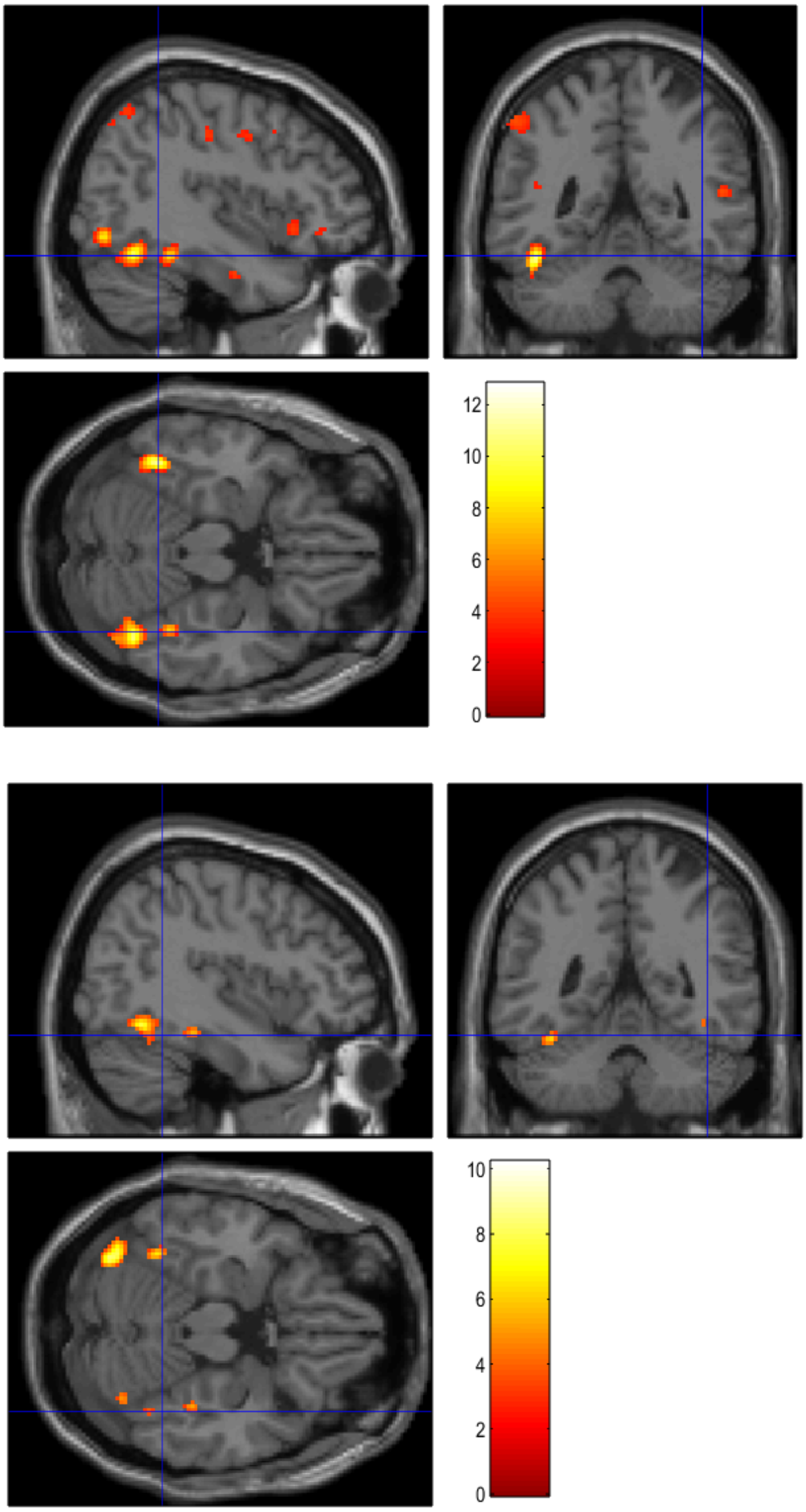

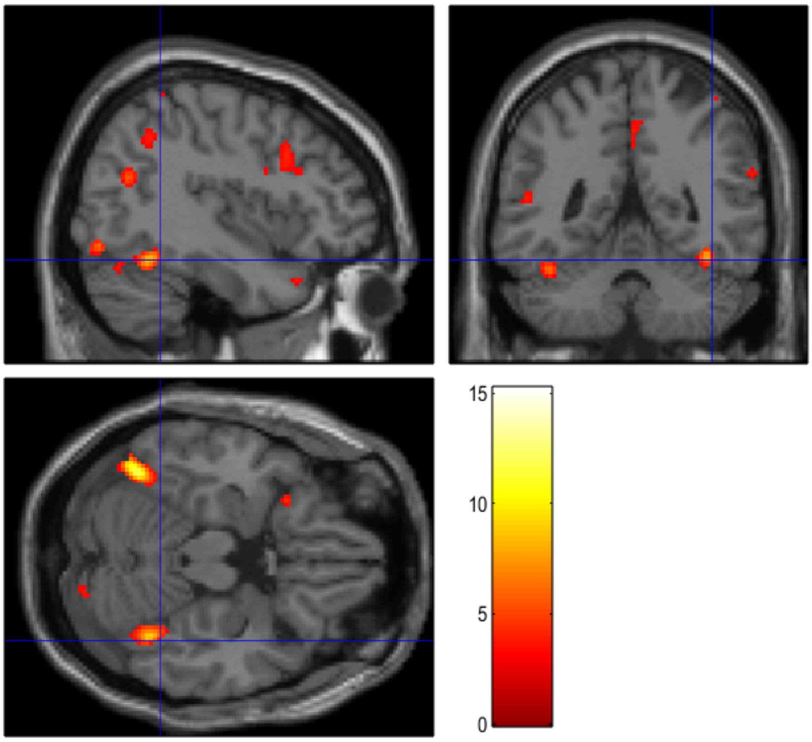

Note. Exemplary SPM activation maps applying the F>O contrast (p=0.001,uncorr.) for three different subjects: sub 08, sub 15, sub 19. MNI coordinates set to [42,-48,-20].

Bayesian Model Comparison

Figure 2: Model Comparison Results

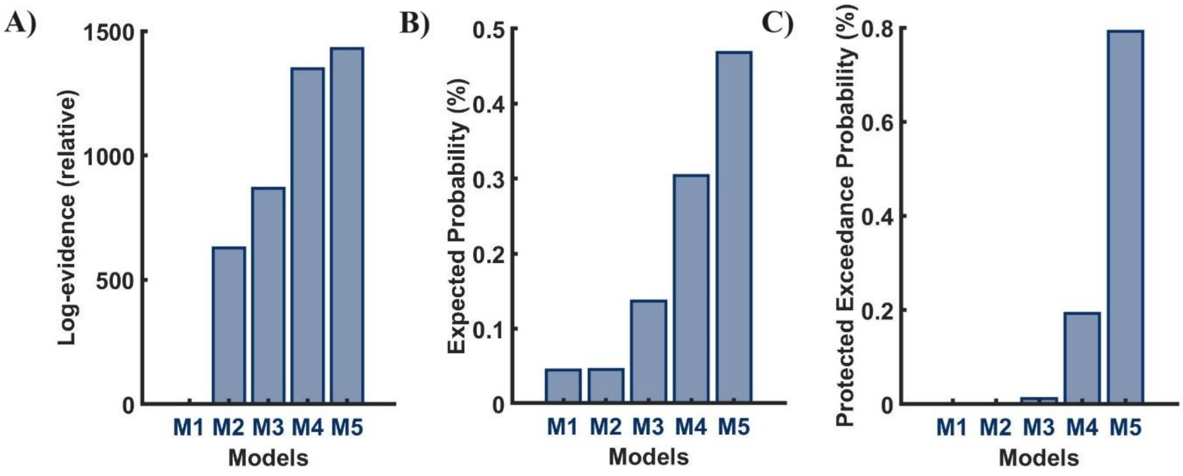

Note. Different BMS Criteria: (A) Model Evidence, (B) Model Posterior Means and C) Protected Exceedance Probability. A) Relative log model evidences approximated by the negative free energy, summed over subjects, and subtracted by the evidence of the lowest model. B) Expected Probability C) Exceedance Probability corrected for the Bayes Omnibus Risk (BOR) of 0.0053.

Figure 3: DCM Quality Assessment

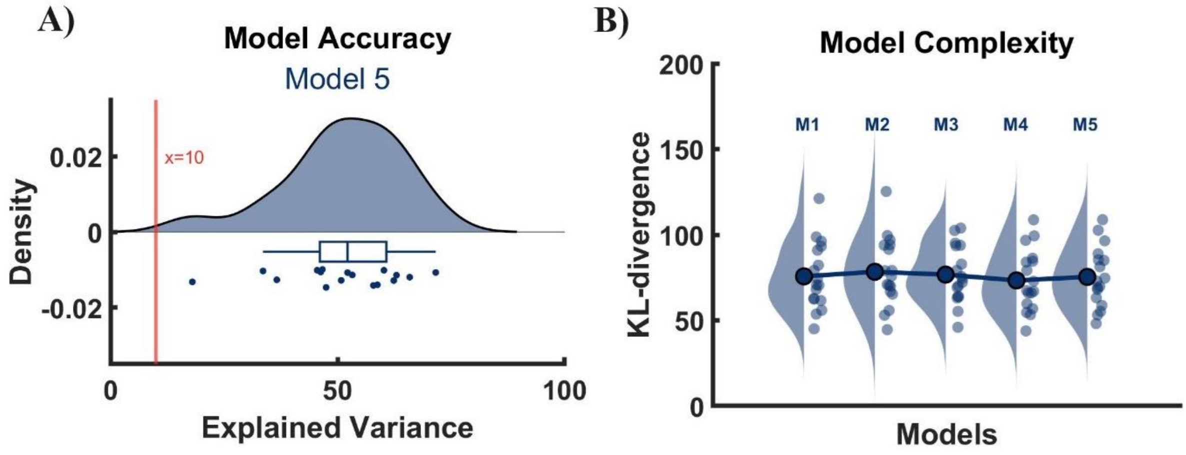

Note. DCM Quality Measures including Model Accuracy (A) and Model Complexity (B). A) Density of explained variance across all participants' model five (M5). Dots indicate individual models' while the red line shows the cut off value for low quality models. B) Kulback-Leibler Divergence between prior and posterior distributions for all five models across each participant.

BMS Validation

Guided by the parameter values of a single participant (sub 20), we chose mean and standard deviations of a normal distribution to select our simulation parameter means and their covariance for the A, B and C-Matrix. This way we constrained our parameter values to a range of naturally occurring numbers, derived from real data, similar to Litvak et al. (Litvak et al., 2019). In short, we randomly selected a combination of parameters for 100 different models for each of our five model types. Subsequently, we used DCM, to derive the timeseries for all six ROIs from the underlying, true models we generated earlier. Now, pretending the true model remains unknown, the same five DCM model types (M1,M2,M3,M4,M5) from our original model space were constructed and estimated using the simulated timeseries data. With the help of random-effects (RFX) BMS we then could extract the best fit model. We applied BMS over a subset of 20 simulations, resampling 100 subsets iteratively across the 100 simulations. This means we selected across the first 20 simulations, then across the second to twentyfirst simulations and so on. After applying BMS we could check how often the BMS returned the true winning model used for data construction. If for 100 model selection procedures, across a specific model type, the correct winning model was found, then BMS was successful.

After running model estimation and model selection of our simulated model space we retrieved the model evidence and the expected probability to determine which model won. For each of our five model types 100 BMS procedures indicate that

most frequently the true winning model is selected. This selection happens to 100% for all models except M4. When M4 models are generated, BMS recovers to 63% M5 as the winning model. Interestingly, M5 simulations have the highest relative log evidence, followed by M4 and M20. Highest deviations in their values is found for M4, least for M1. Further, we found M2 to have the highest mean expected probability (m = 0.84), followed by M1 (m = 0.84) and M5 (m = 0.83). M4 yields the lowest expected probability (m = 0.57) and the smallest relative expected probability (m = 0.25), when comparing its expected probability to the expected probability of the second largest model.

Figure 4: Simulation Evidences

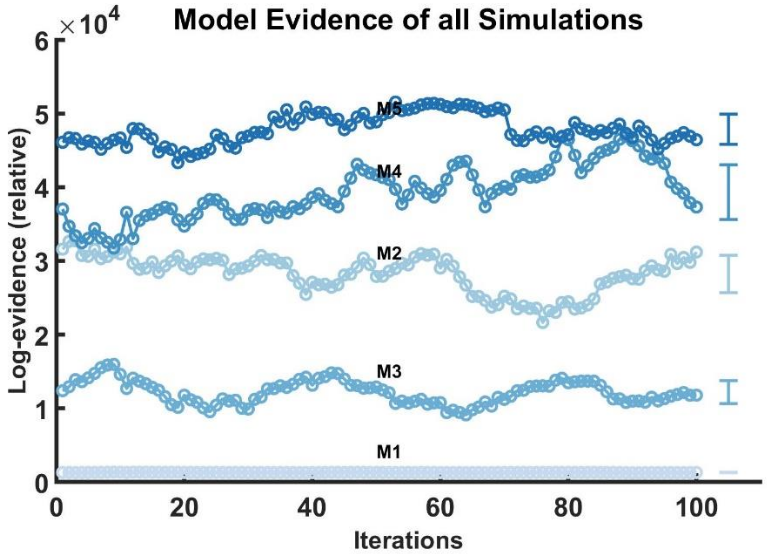

Note. Relative Log-evidence across all iterations of model simulations for each underlying true model (bold text). Calculated as the difference between lowest and highest log-evidence over the possible models.

Parameter Averaging

Table 5: Bayesian Model Averaging Results

Matrix	Description	Mean ± Std	Range	Posterior Probability
A	*Self-Connections*			
	**Left FFA**	**-0.05 ± 0.03**	**[-0.42; 0.32]**	**0.96**
	Right FFA	0.01 ± 0.03	[-0.18;0.38]	0.59
	**Left OFA**	**0.17 ± 0.03**	**[-0.31;0.49]**	**1.00**
	**Right OFA**	**0.21 ± 0.02**	**[-0.19;0.50]**	**1.00**
	**Left V1**	**0.89 ± 0.02**	**[0.63;1.16]**	**1.00**
	**Right V1**	**0.81 ± 0.02**	**[0.28;1.33]**	**1.00**
	Interhemispheric Connections			
	Left FFA -> Right FFA	-0.03 ± 0.02	[-0.24;0.36}	0.94
	**Right FFA -> Left FFA**	**-0.09 ± 0.02**	**[-0.28;0.08]**	**1.00**
	Left OFA -> Right OFA	-0.01 ± 0.02	[-0.35;0.74]	0.78
	**Right OFA -> Left OFA**	**-0.06 ± 0.01**	**[-0.67;0.32]**	**1.00**
	Ipsilateral Connections			
	**Left OFA -> Left FFA**	**-0.11 ± 0.02**	**[-0.53;0.36]**	**1.00**
	**Left FFA -> Left OFA**	**-0.11 ± 0.03**	[-0.92;0.36]	**1.00**
	**Right OFA -> Right FFA**	**-0.13 ± 0.02**	**[-0.46;0.36]**	**1.00**
	**Right FFA -> Right OFA**	**-0.15 ± 0.03**	**[-0.85;0.50]**	**1.00**
	**Left V1 -> Left OFA**	**0.34 ± 0.01**	**[0.10;0.70]**	**1.00**
	**Left V1 -> Left FFA**	**0.12 ± 0.01**	**[-0.08;0.30]**	**1.00**
	**Right V1 -> Right OFA**	**0.40 ± 0.01**	**[-0.11;0.73]**	**1.00**
	**Right V1 -> Right FFA**	**0.20 ± 0.01**	**[-0.15;0.75]**	**1.00**
B	*Interhemispheric Connections*			
	**Central Stimulus on Left FFA -> Right FFA**	**0.27 ± 0.13**	**[-1.18;2.46]**	**0.98**
	**Central Stimulus on Right FFA -> Left FFA**	**0.23 ± 0.13**	**[-1.54;2.99]**	**0.97**
	Central Stimulus on Left OFA -> Right OFA	0.02 ± 0.13	[-1.23;1.55]	0.56
	Central Stimulus on Right OFA -> Left OFA	-0.12 ± 0.10	[-2.21;1.54]	0.87
	Ipsilateral Connections			
	**Central Stimulus on Left OFA -> Left FFA**	**-0.36 ± 0.12**	**[-2.95;1.21]**	**1.00**
	Central Stimulus on Left FFA -> Left OFA	0.12 ± 0.14	[-0.85;3.52]	0.80
	**Central Stimulus on Right OFA -> Right FFA**	**-0.29 ± 0.09**	**[-2.19;1.02]**	**1.00**
	**Central Stimulus on Right FFA -> Right OFA**	**-0.34 ± 0.13**	**[-1.93;0.63]**	**1.00**
	**Central Stimulus on Left V1 -> Left OFA**	**0.68 ± 0.11**	**[-0.53;3.31]**	**1.00**
	**Central Stimulus on Left V1 -> Left FFA**	**0.39 ± 0.09**	**[0.00;2.00]**	**1.00**
	**Central Stimulus on Right V1 -> Right OFA**	**0.43 ±0.09**	**[-1.57;1.64]**	**1.00**
	**Central Stimulus on Right V1 -> Right FFA**	**0.56 ± 0.08**	**[0.00;1.89]**	**1.00**
	Interhemispheric Connections			
	**Left Stimulus on Left FFA -> Right FFA**	**-0.33 ± 0.16**	**[-2.46;1.35]**	**0.98**
	**Left Stimulus on Right FFA -> Left FFA**	**0.38 ± 0.06**	**[0.02;1.16]**	**1.00**
	**Left Stimulus on Left OFA -> Right OFA**	**-0.35 ± 0.14**	**[-2.68;0.91]**	**0.99**
	**Left Stimulus on Right OFA -> Left OFA**	**0.38 ± 0.07**	**[-0.26;1.78]**	**1.00**
	Ipsilateral Connections			
	Left Stimulus on Left OFA -> Left FFA	-0.14 ± 0.09	[-1.82;0.54]	0.93
	**Left Stimulus on Left FFA -> Left OFA**	**0.33 ± 0.14**	**[-1.31;2.00]**	**0.99**
	Left Stimulus on Right OFA -> Right FFA	-0.14 ± 0.10	[-2.01;1.26]	0.92
	Left Stimulus on Right FFA -> Right OFA	0.11 ± 0.15	[-1.81;1.16]	0.77
	Left Stimulus on Left V1 -> Left OFA	-0.00 ± 0.23	[-0.02;0.02]	0.50
	Left Stimulus on Left V1 -> Left FFA	0.00 ± 0.18	[-0.01;0.01]	0.51
	**Left Stimulus on Right V1 -> Right OFA**	**0.65 ± 0.09**	**[-0.33;2.32]**	**1.00**
	**Left Stimulus on Right V1 -> Right FFA**	**0.73 ± 0.09**	**[0.00;2.49]**	**1.00**
	Interhemispheric Connections			
	**Right Stimulus on Left FFA -> Right FFA**	**0.83 ± 0.11**	**[0.16;2.21]**	**1.00**

	**Right Stimulus on Right FFA -> Left FFA**	**-0.42 ± 0.15**	**[-2.91;1.05]**	**1.00**
	**Right Stimulus on Left OFA -> Right OFA**	**0.15 ± 0.04**	**[-0.41;0.93]**	**1.00**
	**Right Stimulus on Right OFA -> Left OFA**	**-0.34 ± 0.13**	**[-1.84;1.48]**	**1.00**
	Ipsilateral Connections			
	**Right Stimulus on Left OFA -> Left FFA**	**-0.30 ± 0.11**	**[-2.26;1.29]**	**1.00**
	Right Stimulus on Left FFA -> Left OFA	-0.10 ± 0.16	[-1.69;1.33]	0.73
	**Right Stimulus on Right OFA -> Right FFA**	**-0.31 ± 0.08**	**[-1.84;0.55]**	**1.00**
	**Right Stimulus on Right FFA -> Right OFA**	**0.32 ± 0.13**	**[-1.47;1.89]**	**0.99**
	**Right Stimulus on Left V1 -> Left OFA**	**0.69 ± 0.08**	**[0.00;1.70]**	**1.00**
	**Right Stimulus on Left V1 -> Left FFA**	**0.50 ± 0.07**	**[0.00;1.61]**	**1.00**
	Right Stimulus on Right V1 -> Right OFA	0.00 ± 0.23	[-0.02;0.08]	0.50
	Right Stimulus on Right V1 -> Right FFA	0.00 ± 0.18	[-0.01;0.02]	0.50
C	*Direct Inputs*			
	**Central Stimulus on Left V1**	**0.67 ± 0.02**	**[0.30;1.23]**	**1.00**
	**Central Stimulus on Right V1**	**0.78 ± 0.02**	**[0.10;1.47]**	**1.00**
	**Left Stimulus on Right V1**	**0.53 ± 0.02**	**[0.07;0.95]**	**1.00**
	**Right Stimulus on Left V1**	**0.58 ± 0.02**	**[0.10;1.06]**	**1.00**

Note. Bayesian Model Averaging (BMA) results across all parameters, including the A-, B- and C-Matrix.

Piloting

Before registration we collected and analysed data from one participant in order to see if the paradigm works satisfactory. This data has been preprocessed and defaced before making it available under the OSF pilot directory of this project. For data analysis, we followed the analysis plan described above to see whether our stimuli activate face-related areas as well as the occipital pole. Because the dataset only contains one participant, group analysis has been discarded. To find ROI activity, we select the closest peaks to group level activities from Thome et al. (Thome et al., 2022). Table 6 shows that for the pilot participant, peak activities for all Regions of Interest could be found.

Table 6: Coordinates and t-values of peak-level activations within Brodmann area 17 (V1) or spheric masks centred around [-42 -86 -10] left OFA, [46 -80 -8] right OFA, [-42 -50 -20] left FFA, [42 -46 -18] right FFA, see Thome et al. (Thome et al., 2022).

ROI	Hemisphere	Contrast	MNI coordinates			Cluster size (voxels)	t-value
			x	y	z		
V1	Left	F+O > baseline	-24	-94	-14	241	16.62
V1	Right	F+O > baseline	22	-96	-8	265	16.45
OFA	Left	F > O	-40	-82	-14	60	7.69
OFA	Right	F > O	40	-74	-8	10	5.92
FFA	Left	F > O	-48	-50	-24	10	5.88
FFA	Right	F > O	46	-44	-22	40	9.83

Lateralization

Table 7: *Hemispheric lateralization of the brain activation pattern elicited by foveal stimulus presentation, as quantified by a lateralization index (LI) for each subject. The LI is separately calculated for the early visual cortex (EVC), the occipital face area (OFA) and the fusiform face area (FFA). Positive LI values indicate left-hemispheric dominance, while negative values reflect right-hemispheric dominance.*

ID	EVC	OFA	FFA
**01**	-0.281	1.000	-0.187
**02**	-0.072	0.312	-0.497
**03**	0.123	0.107	0.111
**04**	-0.050	-0.321	-0.187
**05**	-0.030	-0.613	-0.093
**06**	-0.207	-0.534	-0.491
**07**	-0.151	-0.420	-0.365
**08**	-0.519	0.097	0.209
**09**	-0.038	-0.388	-0.144
**10**	-0.319	-0.070	0.366
**11**	-0.128	0.023	0.010
**12**	-0.021	-0.505	-0.301
**13**	-0.016	1.000	-1.000
**14**	-0.071	1.000	-0.622
**15**	-0.015	-0.418	-0.082
**16**	-0.152	0.329	-0.603
**17**	-0.068	0.000	0.011
**18**	0.042	-0.525	0.026
**19**	-0.328	0.073	-0.339
**20**	-0.038	-1.000	-0.144

Resources

Esteban, O., Birman, D., Schaer, M., Koyejo, O. O., Poldrack, R. A., & Gorgolewski, K. J. (2017). MRIQC: Advancing the automatic prediction of image quality in MRI from unseen sites. *PLOS ONE*, *12*(9), e0184661. https://doi.org/10.1371/journal.pone.0184661

Litvak, V., Jafarian, A., Zeidman, P., Tibon, R., Henson, R. N., & Friston, K. (2019, October). There's no such thing as a 'true' model: the challenge of assessing face validity. In *2019 IEEE International Conference on Systems, Man and Cybernetics (SMC)* (pp. 4403–4408). Retrieved 2023-12-28, from https://ieeexplore.ieee.org/document/8914255 (ISSN: 2577-1655) doi: 10.1109/SMC.2019.8914255

Thome, I., García Alanis, J. C., Volk, J., Vogelbacher, C., Steinsträter, O., & Jansen, A. (2022). Let's face it: The lateralization of the face perception network as measured with fMRI is not clearly right dominant. *NeuroImage*, *263*, 119587. https://doi.org/10.1016/j.neuroimage.2022.119587
